# Nature-Inspired Strategies for Sustainable Degradation of Synthetic Plastics

**DOI:** 10.1021/jacsau.4c00388

**Published:** 2024-08-27

**Authors:** Sreeahila Retnadhas, Daniel C. Ducat, Eric L. Hegg

**Affiliations:** † Department of Biochemistry and Molecular Biology, Michigan State University, East Lansing, Michigan 48824, United States; ‡ MSU-DOE Plant Research Laboratory, Michigan State University, East Lansing, Michigan 48824, United States

**Keywords:** sustainability, synthetic plastics, nature inspired, cellulose degradation, anchor domains, multienzymes scaffold, polyethylene upcycling

## Abstract

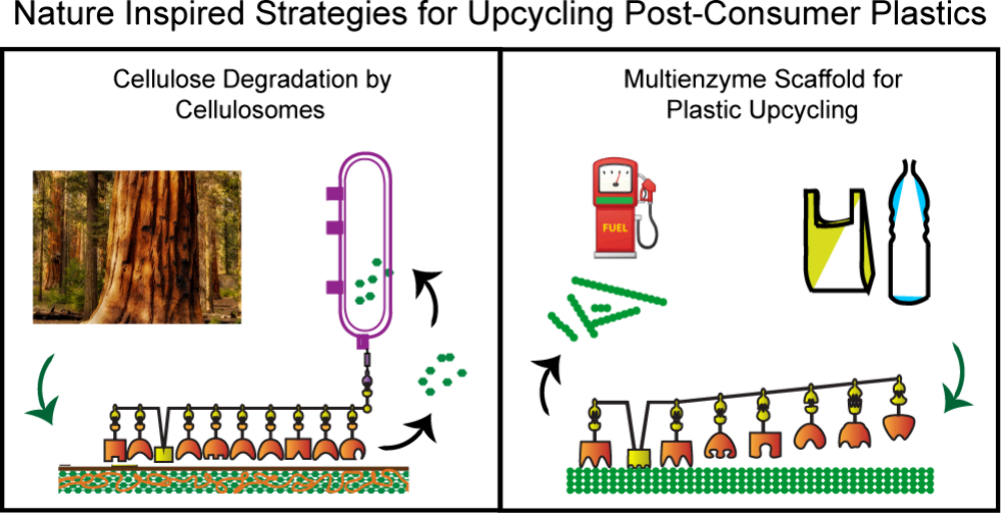

Synthetic plastics have become integral to our daily lives, yet their escalating production, limited biodegradability, and inadequate waste management contribute to environmental contamination. Biological plastic degradation is one promising strategy to address this pollution. The inherent chemical and physical properties of synthetic plastics, however, pose challenges for microbial enzymes, hindering the effective degradation and the development of a sustainable biological recycling process. This Perspective explores alternative, nature-inspired strategies designed to overcome some key limitations in currently available plastic-degrading enzymes. Nature’s refined degradation pathways for natural polymers, such as cellulose, present a compelling framework for the development of efficient technologies for enzymatic plastic degradation. By drawing insights from nature, we propose a general strategy of employing substrate binding domains to improve targeting and multienzyme scaffolds to overcome enzymatic efficiency limitations. As one potential application, we outline a multienzyme pathway to upcycle polyethylene into alkenes. Employing nature-inspired strategies can present a path toward sustainable solution to the environmental impact of synthetic plastics.

## 1 Introduction

Plastics have become integral to our daily lives, substituting for materials like wood, metal, and glass in numerous applications due to their robustness, chemical inertness, hydrophobic attributes, and durability. The global plastic market is predicted to grow from $593 billion in 2021 to $811 billion by 2028, with materials primarily originating from nonrenewable sources, such as fossil-based oil and gas.^1^ Yet the same properties of limited reactivity and stability also limits the environmental degradation of synthetic plastics, which may persist over periods spanning from 50 to 600 years.^2^ The expansion of plastic production since their industrial production in the 1950s, coupled with inadequate waste management practices, has culminated in the accumulation of plastic waste in landfills and aquatic environments. The most commonly produced synthetic plastics in 2021 are polyethylene (PE, 26.9%), polypropylene (PP, 19.3%), polyvinyl chloride (PVC, 12.9%), polyethylene terephthalate (PET, 6.2%), polyurethanes (PU, 5.5%), and polystyrene (PS, 5.3%),^3^ none of which are biodegradable in marine or terrestrial environments. While research into renewable and biodegradable bioplastics has garnered extensive attention, currently bioplastics lack the necessary properties to fully replace traditional plastics in various industries.^4^ Therefore, implementing effective plastic refurbishing (recycling and upcycling) strategies remains a vital element of curbing environmental plastic pollution.^5^

Primary recycling of plastics is a closed loop process that involves reuse of plastic without any modification. Traditional recycling (secondary recycling) of postconsumer plastics uses mechanical methods to produce granulated beads that are reformed in downstream applications. Unfortunately, plastics are susceptible to degradation during traditional recycling processes, impacting their mechanical properties and resulting in a lower value of recycled products. This makes traditional recycling an unattractive option for many industries, and it often necessitates the integration of virgin polymer into the recycled material, making secondary recycling an open-loop system.^6^ Tertiary recycling employs chemical methods (such as chemical recovery and energy recovery) and thermolysis techniques (including pyrolysis, gasification, and hydrogenation) that can reduce polymer degradation and help establish closed-loop processes. While these methods hold promise, they tend to be expensive, have a substantial carbon footprint, involve harsh conditions and toxic catalysts, and generate harmful gases.^7^ It is therefore imperative to identify and develop sustainable technologies to combat plastic pollution.

Biological recycling of plastics is a promising area of research that has the potential to be used in sustainable treatment and the upcycling of plastic wastes. In the last two decades, there has been growing interest in biological plastic recycling following the discovery of microorganisms and invertebrates capable of depolymerizing plastics at moderate reaction conditions without the involvement of toxic solvents and chemicals.^8−11^ Biological methods either use live engineered cells or enzymes to refurbish plastics, and these methods are almost always a multistep process.^12^ Unfortunately, biologically based strategies for plastic recycling generally rely on enzymes that are too slow or pathways that are poorly understood at the mechanistic level. Therefore, such processes are currently too inefficient to be employed at scale and remain economically noncompetitive with existing processes for plastic breakdown or recycling. The inefficiency of plastic-degrading enzymes reported in the literature is likely a function of multiple factors, including the following: (1) synthetic plastics are non-native substrates for microbes in the environment; organisms have only been exposed to synthetic plastics in the last 80 years, and enzymes are likely still evolving to optimize their activity; (2) plastics are hydrophobic, making it difficult for polar enzyme surfaces to bind and catalyze degradation reactions; (3) the highly crystalline nature of plastic polymers makes it difficult for enzymes to reach the scissile bonds; and (4) heterogeneous catalysis is generally less efficient due to less surface area of substrates available for the enzyme to bind. Protein engineering is one strategy that has received considerable attention to overcome these limitations (see section 3); we argue herein that alternative rational approaches may assist in overcoming these challenges.

Evolution has identified efficient strategies for degrading natural polymers such as cellulose, cutin, and chitin that have properties similar to those of some synthetic plastics. Strategies for enzymatic-based plastics refurbishing may benefit from bioinspired approaches based on nature’s optimization of the breakdown of natural recalcitrant polymers. While this Perspective briefly reviews recent developments in plastic-degrading enzymes to provide sufficient context, a number of other excellent comprehensive review articles are available on this topic.^13−16^ In this Perspective, we therefore emphasize potential alternative approaches that are not currently in widespread use in the rapidly growing area of plastics degradation but which may nonetheless have promise based on their demonstrated capabilities on improving the breakdown of recalcitrant polymers found in nature. These bioinspired strategies include the following: (1) aforementioned protein engineering to improve enzyme activity and thermal stability (accelerating evolution in the lab); (2) fusion of anchor modules to the enzymes to improve accessibility of hydrophobic substrates to the enzyme (inspired from cellulosome and chitin-degrading enzymes); (3) multienzyme scaffolding strategies (inspired from cellulosomes) to efficiently degrade or upcycle crystalline polymers.

## 2 Enzymes Involved in Plastic Degradation

Microorganisms generally degrade plastics by secreting enzymes that break polymers into short oligomers or monomers that can enter the cell either through a specialized transporter or by diffusion. This section gives a brief overview of the type of enzymes identified to degrade synthetic plastics. These secretory enzymes can be broadly divided into two categories: (1) hydrolytic enzymes for hydrolyzable plastics and (2) oxidizing enzymes for nonhydrolyzable plastics.

### 2.1 Hydrolytic Enzymes

Three major hydrolyzable plastics include PET, polyamides,^17^ and PU.^18^ In this Perspective, we focus on hydrolytic enzymes that degrade PET, in part because significant progress has already been achieved in the enzymatic depolymerization of PET. Research on enzymes identified for PET hydrolysis has provided valuable instructional examples illustrating how similar strategies could lead to advances in processing other recalcitrant plastic polymers. PET shares a similar backbone structure with certain naturally occurring polyesters, such as cutin. Enzymes like cutinases, lipases, and esterases, known for their versatile activities in breaking down natural polyesters, have demonstrated the capability to hydrolyze PET. Furthermore, specific enzymes (PETases) that target aromatic polyesters have been identified during the past decade and have been shown to have considerable potential in degrading PET.^14^

#### 2.1.1 Cutinases, Lipases and Esterases

These enzymes belong to a broader class of carboxyl ester hydrolases (serine hydrolases) and have a α/β hydrolase fold with a Ser-His-Asp catalytic triad as their active site amino acids. Cutinases are secreted by phytopathogenic microorganisms (fungi and bacteria) to help in the degradation of the protective cutin (natural polyester) layer of plants. Most of the cutinases have a shallow and open substrate binding cleft which is important to hydrolyze long chains of cutin polyester.^19^ Due to higher crystallinity and bulkier aromatic composition of PET when compared to aliphatic cutins, cutinases were initially identified to cause only surface hydrolysis of PET by targeting chain ends and loops protruded from the surface instead of degrading inner blocks of PET materials.^20^ Enzymes from thermophilic bacteria have been the center of research on PET-degrading cutinases, as the high glass transition temperature of PET (*T*_g_ ∼ 70 °C) necessitates a hydrolysis reaction compatible with these higher temperatures for efficient catalysis. A cutinase, TfH, from the thermophilic actinomycete *Thermobifida fusca*, was the first enzyme found to degrade PET.^21^ Since then, several bacterial cutinases such as Tfu_0882,^22^ TfCut1,^22^ TfCut2,^22^ and Thf42_Cut1^23^ from different strains of *T. fusca*, Thc_Cut1^23^ and Thc_Cut2^23^ from *T. cellulosilytica*, and Tha_Cut1^24^ from *T. alba* DSM431185 were found to either modify the surface^23,24^ or hydrolyze PET to monomers.^22^ A thermostable cutinase isolated from leaf compost, leaf compost cutinase (LCC), was shown to hydrolyze PET^25^ and has been popular for protein engineering studies due to its thermal stability and efficient hydrolase activity.^26,27^

Lipases catalyze the hydrolysis of water-insoluble long-chain triglycerides into fatty acids and glycerol. Interestingly, some lipases can hydrolyze the PET surface despite having closed active site structures with lid domains.^20^ Esterases, on the other hand, naturally break down simple esters to produce more bioavailable carboxylic acids and alcohols. Certain esterases, such as glucuronoyl esterase, acetylxylan esterase, *p*-coumaroyl esterase, and ferulic acid esterase, play a role as auxiliary enzymes in lignocellulosic material degradation by hydrolyzing ester bonds between lignin and carbohydrates.^28^ The open active site structures of many esterases, such as Est119^29^ and Est1 from *Thermobifida alba* AHK119 and Thh_Est^30^ from *Thermobifida halotolerans*, allow them to modify the surface of PET. Nonetheless, the rate of PET degradation via lipases and esterases tends to be lower in comparison to cutinases, possibly due to their more polar and deeper active site structures. There are a number of excellent reviews that provide greater detail on PET-modifying esterases, lipases, and cutinases.^14,20,31,32^

#### 2.1.2 PETases

**Figure 1:**
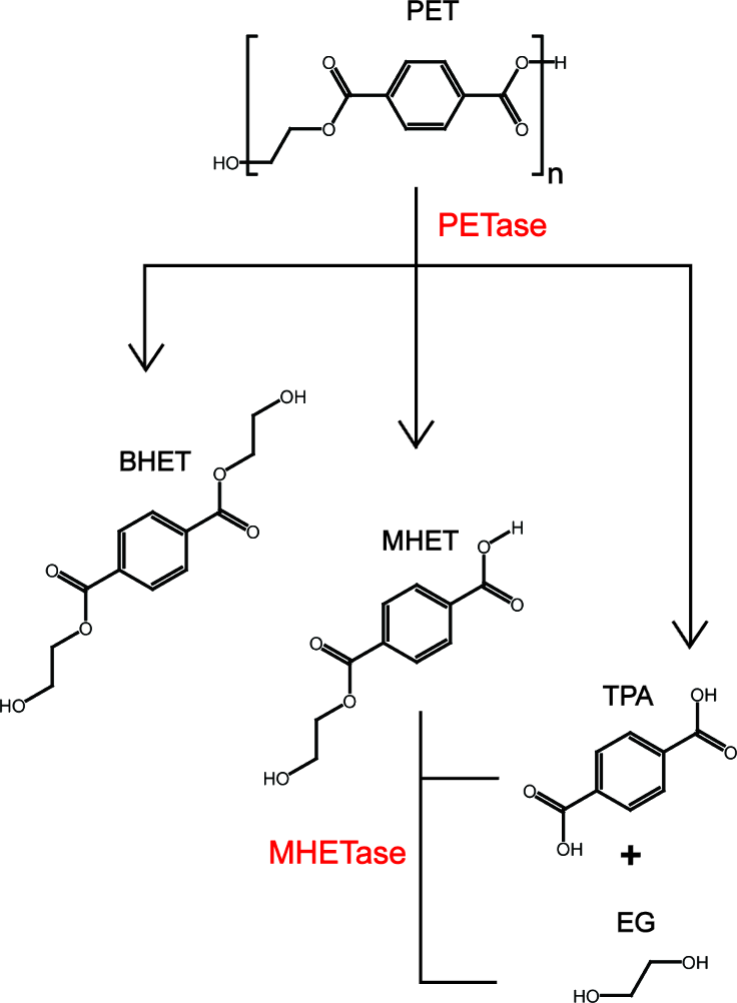
Hydrolysis of PET by enzymes secreted by PET-degrading bacteria: PETase catalyzes the hydrolysis of PET into mono(2-hydroxyethyl) terephthalic acid (MHET as the major product), bis(2-hydroxyethyl) terephthalate (BHET), terephthalic acid (TPA), and ethylene glycol (EG). MHETase further hydrolyzes MHET into TPA and EG, which can be readily catabolized by the cells.

Ever since the discovery of a secreted PET hydrolase from *Ideonella sakaiensis* (*Is*PETase) in 2016,^33^ this enzyme has served as a focal point of research in biological plastic degradation processes along with the cutinase, LCC. In the original paper, *Is*PETase was reported to break down PET into a major product, mono(2-hydroxyethyl) terephthalic acid (MHET), and three minor products, terephthalic acid (TPA), ethylene glycol (EG), and bis(2-hydroxyethyl) terephthalate (BHET). MHETase further hydrolyzes MHET into TPA and ethylene glycol (EG), which are directly utilized for cellular growth (Figure 1).^33^*Is*PETase belongs to the α/β hydrolase superfamily with close structural similarity to the cutinase TfH from *T. fusca*, which also has some limited PETase activity.^21^ Notably, the active site of *Is*PETase is wider than that of TfH, facilitating PET substrate binding for enhanced catalysis.^34,35^ While there are hypotheses suggesting that this enzyme has evolved from polycyclic aromatic hydrocarbon-degrading enzymes and cutinases,^36,37^ it is important to recognize that the nomenclature distinctions between esterases, cutinases, and PETases are not clearly defined, raising the possibility that *Is*PETase is simply a promiscuous cutinase.^20^ Recent research to prospect and characterize PET hydrolases from multiple sources has been comprehensively reviewed elsewhere,^13,38,39^ and these efforts have expanded the enzyme pool for understanding degradation mechanisms and advancing efficient PET processing technologies.

### 2.2 Oxidizing Enzymes

Unfortunately, most of the fossil fuel-based plastics (∼60% of the total plastics synthesized)^3^ are nonhydrolyzable due to their inert C–C backbone. Several PP-, PE-, and PS-degrading microorganisms and insect larvae have been identified from plastic-contaminated environments.^10,40,41^ Typically, enzymes that catalyze the oxidation of these plastics are necessary to lay the groundwork for subsequent depolymerization by making the inert C–C backbone more reactive. In the environment, the oxidation processes catalyzed by secreted enzymes are supported by abiotic factors, such as radiation and heat. Short-chain intermediates released following polymer fragmentation are proposed to be taken up by the microorganisms for assimilation and mineralization.^2,42^ Classes of such plastic-oxidizing enzymes are broad and include (a) monooxygenases such as cytochrome P450 monooxygenases and alkane hydroxylases, (b) insect hexamerins, and (c) other ligninolytic enzymes such as laccases, peroxidases, and peroxygenases.

#### 2.2.1 Monooxygenases

The cytochromes P450 (CYP450s) and alkane hydroxylases (AHs) are two monooxygenases that have promising potential in PE degradation cascades. CYP450s are heme-thiolate proteins that are capable of oxidizing C–H bonds in diverse substrate molecules. These enzymes are generally membrane bound and require a redox partner to transfer two electrons from a donor, such as NADH. CYP450s have been found to catalyze the hydroxylation of both the terminal (ω) and internal alkyl groups of *n*-alkanes, fatty acids, and fatty alcohols, producing their corresponding oxidation product. The ability of CYP450s to catalyze the hydroxylation of both terminal and internal alkyl groups makes them an attractive option to use in PE-degrading cascades.^43^ Purified CYP102A5.v1 from *Bacillus thuringiensis* JNU01 exhibited PE-oxidizing property as indicated in a recent report based on the increased number of oxygen-containing groups in enzyme-treated PE when compared to untreated PE and the ability of CYP102A5.v1 to oxidize NADPH when PE is used as the substrate.^44^ This experimental evidence of a purified P450 enzyme oxidizing untreated PE provides a platform for enzyme engineering to enhance its activity.

AHs are nonheme iron-containing monooxygenases that catalyze the hydroxylation of the terminal alkyl group in alkanes, which helps in further oxidation and degradation of the chain by β-oxidation.^45^ As a result, these enzymes are widely used in the bioremediation of hydrocarbon contamination.^46^ There have been reports of these enzymes facilitating PE degradation,^47,48^ but it is possible that they are simply oxidizing linear alkanes in the PE substrate formed during pyrolysis. Nonetheless, they are promising enzymes to be used in PE upcycling cascades to facilitate functionalizing fragmented PE as proposed elsewhere.^43^

A screening method based on a Hidden Markov Model identified HIS1, a dioxygenase from rice, as a target for the oxidative degradation of PP.^49^ HIS1 belongs to the Fe(II)/2-oxoglutarate-dependent oxygenase family with structural similarity to a catalase-peroxidase from *Aspergillus nidulans* FGSC A4 (Uniprot ID: Q96VT4) that was reported to have PVC degradation activity.^50^ Cracks, pores, and fragmentation on the surface of the PP film were induced by HIS1 treatment, as revealed by atomic force microscopy and scanning electron microscopy. Further, the presence of hydroxyl groups and double bonds in the enzyme-treated PP film was indicated by Fourier-transform infrared spectroscopy (FTIR), while gel permeation chromatography (GPC) revealed a reduction in the average molecular weight of the treated PP. Gas chromatography–mass spectrometry (GC-MS) further substantiated degradation of PP with the identification of 2-pentene, undecane, dodecane, nonane, trimethyldecane, and pentadecane in the enzyme-treated PP sample.^49^ To the best of our knowledge, this is the only report in the literature that provides experimental evidence of PP degradation with a purified enzyme. The identification of this enzyme offers a starting point for protein engineering efforts aimed at enhancing its PP degradation activity, paving the way for the development of sustainable PP degradation technologies.

#### 2.2.2 Prophenoloxidases/Hexamerin

Enzymes from both the gut microbiome and saliva of *Galleria mellonella*(51) were found to contribute to PE metabolism by facilitating oxidation and fragmentation. To our knowledge, this is one of the two instances where purified enzymes have been shown to induce oxidation and fragmentation of untreated PE.^52^ Cryoelectron microscopy of the insect saliva and subsequent PE oxidation studies helped identify three PE-oxidizing enzymes belonging to the hexamerin/prophenoloxidase family, namely, Demetra, Ceres, and Cora.^53^ These proteins are members of the type-3 copper-containing proteins with two active site copper atoms, and they are generally acknowledged for their capacity to oxidize phenolic compounds (phenoloxidases) and to bind oxygen (hemocyanins). The identification of phenolic compounds, possibly utilized as additives during PE synthesis, in liquid chromatography–mass spectrometry (LC-MS) analysis of treated PE beads led the researchers to hypothesize that these phenolics could be the target of insect prophenoloxidases, generating free radicals (perhaps similar to the laccase mediated system as discussed below) that could cause PE autoxidative chain reaction.^52^ However, the reported presence of copper ions in a position unrelated to hexamerin/prophenoloxidase,^53^ along with the absence of phenoloxidase activity in Ceres,^54^ makes it difficult to predict the reaction mechanism.

It is important to note that a recent preprint by Stepnov et al. is critical of the reported PE-oxidizing activities of insect hexamerins, as they were unable to replicate the PE-oxidizing activities of one of the three reported insect hexamerins, Ceres.^54^ This underscores the need for standardized criteria for both the enzyme and the substrate that must be met before an enzyme can be identified as a plastic-degrading/oxidizing enzyme, as proposed by Lear et al.^55^

#### 2.2.3 Ligninolytic Enzymes

Ligninolytic oxidative enzymes, including laccases, manganese peroxidases (MnP), lignin peroxidases (LiP), and unspecified peroxygenases (UPO), have demonstrated their potential to aid in the degradation of PE through oxidative processes. These enzymes, referred to as lignin-modifying enzymes, engage in the modification of lignin, a process that facilitates its degradation by auxiliary lignin-degrading enzymes.^56^ Laccases are members of the multicopper oxidase (MCO) family that have a characteristic cupredoxin-like fold in their structure and at least four different copper atoms in three copper centers. MCOs that can oxidize the natural substrate of a true laccase, urushiol, have been classified as laccases.^57^ Due to their low substrate specificity, laccases are able to oxidize a wide range of both phenolic and nonphenolic substrates concomitant with the four-electron reduction of O_2_ to water, although their ability to oxidize nonphenolic substrates depends on their respective redox potentials.^58^ In laccase-mediator systems (LMS), low molecular weight phenolic compounds are rapidly oxidized by laccase to generate a low molecular weight radical intermediates that can oxidize high molecular weight target substrates.^59^ One LMS was effectively applied to biodegrade PE and nylon, reducing the average molecular weight of the polymer and forming various oxygen-containing products (aldehydes, alcohols, and ketones).^59,60^ On the basis of upregulated laccase expression (as determined by increased mRNA levels) and increased secretion during the growth of fungal strains on different forms of PE, laccases were indirectly associated with PE oxidation.^61−65^ The presence of oxidized groups and structural changes on enzyme-treated low-density PE films was shown to indicate that a thermophilic laccase can degrade PE.^66^ Despite these reports, there is currently a gap in the literature concerning studies demonstrating the oxidative fragmentation of untreated PE.

Laccase-like multicopper oxidases (LMCOs) constitute another group of enzymes classified within the family of MCO. LMCOs have a type I (T1) copper center that oxidizes substrates and transfers electrons to the trinuclear copper center (T2/T3), where the enzyme reduces O_2_ to water. LCMOs differ from laccases in their substrate specificity. A promising report published recently shows oxidative fragmentation of untreated LDPE powder by two purified enzymes, LMCO2 and LMCO3, from *Rhodococcus opacus* R7. GC-MS analysis of enzyme treated PE powder revealed the presence of carboxylic acids (C8–C18) as the major product, with alcohols (C10), ketones (C11–C19), and alkanes (C12–C30) as minor products.^67^ Considering the laccase reaction mechanism and DFT calculations, the authors proposed that the oxidation of C–H bonds takes place at the T1 copper center, resulting in the formation of a radical aliphatic species. This radical species can then undergo a series of oxidation steps, leading to the formation of alcohols and carbonyls. To the best of our knowledge, this is the only report in the literature demonstrating the oxidative fragmentation of PE by multicopper oxidases with purified enzymes. These enzymes have tremendous potential to be used in the development of sustainable enzymatic PE upcycling technologies.

Peroxidases belong to a group of heme-containing oxidoreductases that catalyze H_2_O_2_-dependent oxidation of several phenolic and nonphenolic substrates. While the involvement of ligninolytic peroxidases such as MnP and LiP in PE oxidation is reviewed in detail elsewhere,^42^ the scarcity of literature on their PE oxidation property with purified enzymes limits their consideration for the development of a PE degradation pathway. Hydroquinone peroxidase from a lignin decolorizing bacterium *Azotobacter beijerinckii* HM121 was reported to degrade water insoluble PS (molecular weight: 930 kDa) in a two-phase system (water:dichroromethane) to water-soluble products within 5 min,^68^ although there has been limited follow-up analysis published since this report. A heme-thiolate enzyme, UPO, functionally represents a hybrid of cytochrome P450s and peroxidases, as their hydroxylation activities are supported by H_2_O_2_ without requiring redox cofactors and electron shuttling proteins. The ability of UPOs to catalyze the oxidation of alkanes at multiple positions (internal and terminal) to produce different combinations of alcohols and carboxylic acids can be exploited for a PE upcycling cascade.^69^

## 3 Protein Engineering To Accelerate Enzyme Evolution

Most existing enzymes do not operate at their maximum capability in vitro (i.e., a rate at which the reaction is limited by diffusion of substrates),^70^ creating a substantial opportunity for enhancing their performance through laboratory-based protein engineering. This holds particularly true for plastic-degrading enzymes, which have evolved in response to different organic materials and have had limited time (<80 years) to optimize their efficacy against synthetic plastics. There are two approaches for protein engineering: directed evolution and rational engineering. The success of directed evolution depends on the ability to efficiently screen large libraries of variants for a required trait. With the availability of structural data, structure prediction tools, and a variety of computational techniques for simulating mutational impacts, rational engineering has gained traction in recent years. As discussed earlier, our understanding of hydrolytic enzymes that break down ester backbones surpasses our knowledge of oxidizing enzymes that enable the degradation of nonhydrolyzable plastics, and therefore most of the protein engineering efforts thus far have focused on the enzymes degrading the hydrolyzable plastic PET. This section focuses on those efforts, specifically in terms of (1) increasing the thermal stability of the enzyme, (2) modifying the substrate binding cleft, (3) alleviating product inhibition, and (4) improving the interaction between polymer and the enzyme sites away from the active site.

### 3.1 Thermal Stability

Due to the high *T*_g_ of PET, its degradation is generally more efficient at higher temperatures. Conversely, high-temperature enzymatic processing of PET is severely limited by the low stabilities of PET hydrolyzing enzymes. Some of the common structural features of naturally occurring thermostable enzymes were incorporated into PET hydrolyzing enzymes by protein engineering to improve their thermal stability as summarized in Table 1, including (a) increased protein compactness, (b) reduced enzyme flexibility, and (c) stronger intramolecular interactions due to increased numbers of disulfide bonds, electrostatic interactions, hydrophobic interactions, and hydrogen bonding.^79^ The variability in PET materials and reaction conditions across different reports makes it challenging to cross-reference studies to identify the most efficient variants. Nonetheless, DepoPETase, generated by directed evolution and shown to have 1400-fold greater activity than *Is*PETase, represents a notable achievement in engineering PET hydrolases.^75^

**Table 1:** Notable Protein Engineering Efforts on PET Hydrolases

mutant	enzyme engineered	amino acids mutated	effects on protein structure	*T*_m_	improvement in PET hydrolysis rate
Thermo-PETase^71^	*Is*PETase	S121E/D186H/R280A	stabilizing central β-sheet in the α/β hydrolase fold	57.62 °C	14-fold at 40 °C
FAST-PETase^72^	Thermo-PETase	N233 K/R224Q/S121E	additional salt bridge and hydrogen bond	67.4 °C	38-fold at 50 °C
Hot-PETase^73^	Thermo-PETase	18 mutations	additional disulfide bond and better central β-sheet packing	82.5 °C	∼27-fold at 65 °C (estimated from Figure 2b of original research article)
Dura-PETase^74^	*Is*PETase	S214H/I168R/W159H/S188Q/R280A/A180I/G165A/Q119Y/L117F/T140D	introduced electrostatic interaction and hydrogen bonding, improved hydrophobic packing, and reduced conformational entropy	77 °C	300-fold at 37 °C
Depo-PETase^75^	*Is*PETase	T88I/D186H/D220N/N233 K/N246D/R260Y/S290P	stabilized active site loops	69.38 °C	1400-fold at 50 °C
Turbo-PETase^76^	*Bhr*PETase	H218S/F222I/A209R/D238 K/A251C/A281C/W104L/F243T	introducing a disulfide bond, and optimizing charge–charge interactions on the protein surface	84 °C	4-fold at 65 °C
ICCG^77^	LCC	F243I/D238C/S283C/Y127G	introducing a disulfide bond	94 °C	1.5-fold at 72 °C
PES-H1 (L92F/Q94Y)^78^	PES-H1	L92F/Q94Y	based on DuraPETase mutation	78.2 °C	2.3-fold at 72 °C

### 3.2 Active Site Modifications

It is logical that cutinases possess broader and more accessible active sites compared to lipases and esterases due to the differences in their natural substrate size. Analogously, PET-hydrolyzing enzymes generally possess wider active site clefts for accommodating bulkier aromatic polyesters like PET.^34,80^ Consequently, protein engineering to increase the size and hydrophobicity of substrate binding clefts has shown success at improving PET hydrolytic activity in LCC (increased volume),^81^ TfCut2 (increased volume and hydrophobicity),^82^ PET hydrolase from the marine bacterium *Pseudomonas aestusnigri* VGXO14^*T*^ (increased volume),^83^ Tfu_0883 from *T. fusca* (increased volume and hydrophobicity),^84^ and ICCG (increased volume).^85^ Furthermore, researchers inspired by the cutinase architecture generated a mutant of *Is*PETase with a narrower substrate binding cleft, and this mutant demonstrated improved PET degradation. Unexpectedly, the improved activity was not due to the change in the volume of the active site but rather due to improved binding of the enzyme on the PET surface.^34^

### 3.3 Product Inhibition

Some of the released products of PET breakdown compete for binding in the enzyme’s active site. MHET, the major water-soluble intermediate of PET hydrolysis, acts as an inhibitor due to its strong affinity to TfCut2 and the low MHETase activity of TfCut2.^86^ Process modification like using a membrane filter reactor to remove MHET during catalysis was shown to improve the PET hydrolysis rate.^87^ Rational engineering by comparing the substrate binding cleft of LCC with TfCut2 resulted in a mutant with improved PET hydrolytic activity because of a 5.5-fold reduction in affinity of the enzyme to MHET.^82^

### 3.4 Surface Interaction with PET

In the context of enzymes catalyzing the transformation of hydrophobic substrates, the interaction between the substrate and enzyme represents one of the limiting factors for efficient catalysis. This challenge becomes even more pronounced when dealing with hydrophobic, bulky, and water-insoluble materials such as synthetic plastics. Natural evolution overcomes this challenge by incorporating binding domains to anchor the enzyme onto the substrate,^88,89^ and this strategy is discussed in detail in later sections. Mutational studies that affect the surface properties of the enzymes have also been shown to enhance the catalytic activity by improving substrate binding. For instance, a mutant of *Is*PETase increases enzyme activity by stabilizing the interaction of Y58 with the substrate.^90^ Reducing surface charge and increasing surface hydrophobicity in the cutinase *Thc*_cut2 improved enzyme activity by increasing its capacity to anchor to PET surfaces.^91^ Interestingly, reducing hydrophobicity around the substrate binding cleft of LCC was found to decrease intermediate accumulation (BHET and MHET) during PET hydrolysis due to reduced binding on the PET solid surface, thereby enabling improved access of the soluble intermediates to the active site.^92^

Ultimately, extensive engineering of PETases has generated several highly active and thermostable variants. Carbios, a French company founded in 2011, has successfully demonstrated the use of their enzyme, ICCG, to depolymerize ∼90% of postconsumer PET flakes within 14 h.^93^ A recent comparative study evaluating the efficiency of four engineered PET hydrolases in the literature for industrial applications based on enzyme properties (stability, catalytic efficiency of enzymes, enzyme compatibility with heterologous expression) and depolymerization reaction (yield and composition of products)^93^ has emphasized the suitability of ICCG over other engineered PETases in degrading postconsumer PET flakes. A separate report demonstrated that TurboPETase, which is not considered in the previous study, is more efficient than ICCG under industrially relevant conditions.^94^ It is important to note that ∼98% degradation of pretreated PET was achieved by ICCG and TurboPETase under different conditions in the respective reports, and ICCG has been shown to be superior to other enzymes in catalyzing PET hydrolysis to its monomers without forming intermediates that might require additional enzymes for complete depolymerization to monomers.

## 4 Nature-Inspired Strategy for Enzymatic Degradation of Synthetic Plastics

Enzymes constitute just one facet of naturally occurring polymer-degrading biological systems that can be repurposed for the degradation of synthetic polymers, and the strategies utilized by these biological systems offer inspiration for the development of new plastic-degrading processes. Cellulose, composed of linear chains of glucose units connected by β-1–4 glycosidic bonds, is the primary component of plant cell walls, providing both structure and strength to the plant. Just as importantly, however, this polymer also acts as the primary line of defense against plant pathogens and grazers by providing a physical barrier that is also resistant to enzymatic attack. Similar to the linear alkyl chains in PE and PP that form highly crystalline structures due to strong van der Waals forces, cellulose also forms highly crystalline structures, in this case due to multiple strong hydrogen bonds between the glucose units that cause the individual glucose chains to arrange into linear microfibrils. These microfibrils exhibit a highly crystalline structure that contribute significantly to the mechanical robustness of plant cell walls and resist enzyme accessibility.^95^ Chitin (β-(1–4)-poly-*N*-acetyl-d-glucosamine) has a structure similar to that of cellulose and is present both in the cell walls of fungi and in the exoskeletons of shrimps, crabs, and crustaceans. All of these polymers are further reinforced by “additives” that serve to modulate physical features, cross-link strands, and increase the chemical complexity of the polymer and increase its recalcitrance to modification. Unlike plastics, however, both cellulose and chitin have been present in nature for millions of years, and correspondingly sophisticated enzyme systems have evolved to degrade involving the participation of multiple enzymes.

**Figure 2:**
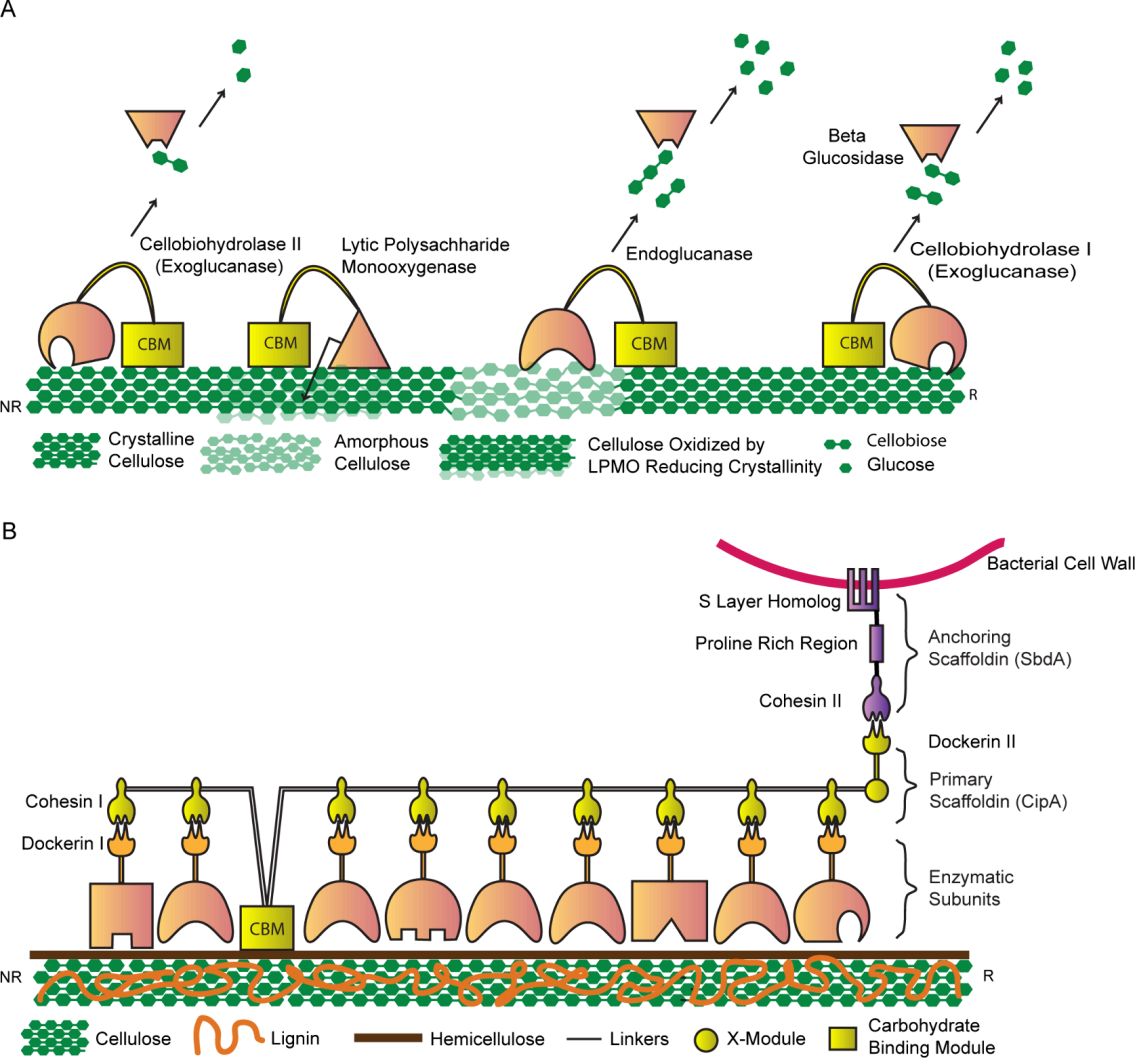
Enzymatic degradation of cellulose. (A) Involvement of separable enzymes with substrate binding domains that promote complete degradation of crystalline cellulose: exoglucanases I and II attack from the reducing and nonreducing ends of cellulose fibers, respectively, to release cellobiose; LPMOs oxidize crystalline regions of cellulose to make them amorphous, which is hydrolyzed by endoglucanases to release cellobiose; and finally, glucose units are released from cellobiose by β-glucosidases. (B) A simple model representing cell wall-bound cellulosome in *Clostridium thermocellum*: Scaffoldin subunit (CipA) consists of nine type I cohesin modules (CohI), a type II dockerin module (DocII), a carbohydrate binding module (CBM) and an X-module (helps in scaffold stability), all fused together by linkers. Nonspecific interactions between CohI of CipA and DocI of enzymatic subunits allow for variability in the composition of enzymes linked. CohII, which is linked to the SLH domain (in SdbA, an anchoring scaffoldin) that binds cell wall components and interacts with DocII of CipA subunit, thereby facilitating cell surface anchorage of the whole cellulosome. (This original figure is inspired from Xu et al.^96^).

Two common features among evolved enzyme systems responsible for degrading natural polymers involve (a) the targeted positioning of enzymes proximal to their substrate and (b) coordination of multiple enzymes simultaneously to achieve more complete degradation (Figure 2A). In the most straightforward of these strategies, most enzymes in the system possess both a catalytic domain and a binding domain that directly recognize the target substrate.^97^ For example, endoglucanases, which hydrolyze internal glycosidic bonds within cellulose chains to generate shorter chains and produce additional terminal ends, typically have one or more carbohydrate binding modules (CBM) fused to their processive catalytic subunit.^98^ A similar strategy has been well-documented in many lytic polysaccharide monooxygenase (LPMO) enzymes that oxidatively cleave glycosidic bonds in crystalline regions of cellulose and are recruited to cellulose via a separate protein domain that helps anchor the catalytic subunit to the cellulose. Collectively, targeting these enzymes to their substrates helps increase their activity due to proximity effects (i.e., increasing the effective concentration of the enzyme near the substrate), which may be particularly important for enzymes acting on crystalline solid substrates.

Some anaerobic bacteria and fungi have evolved more sophisticated methods of efficient cellulose degradation via positioning and coordinating multiple cellulolytic enzymes on a common protein scaffold (Figure 2B). These multienzyme scaffolds, referred to as cellulosomes, are important for the growth of cellulose degraders on crystalline cellulose, presumably by improving the synergistic activities of cellulases by their collective arrangement into the complex with a CBM.^99^ Cellulosomes consist of two primary components: the noncatalytic structural proteins, specifically the cohesin-containing scaffoldins, and the diverse catalytic elements that possess dockerin modules. Scaffoldins are composed of multiple type-I cohesin units separated by linkers, and the assembly of cellulosomes is facilitated by the strong affinity between type-I cohesion modules of scaffoldins and type-I dockerin modules of enzymes.^100^ The interaction between the type-II dockerin of scaffoldin and type-II cohesin fused to the SLH domain facilitates the anchoring of cellulosomes to the bacterial cell by engaging the SLH domain with the cell wall components (Figure 2B). A mutant of *Clostridum thermocellum* with defective production of scaffoldin demonstrated comparable hydrolytic activity against soluble β-glucans but displayed 15-fold less hydrolytic activity against crystalline cellulose compared to the wildtype.^101^ This observation is supported by an AFM study demonstrating that cellulosomes morph their shapes according to the shape of substrate surface and hydrolyze the material beneath them whereas free cellulases peel off crystalline cellulose while sliding along the substrate.^102^

Cellulosomes are gaining significance in the field of biofuel production, where they are used to generate highly fermentable sugars from lignocellulosic biomass, as well as from the organic fraction of municipal solid waste (OFMSW). It was reported in the final report summary of “The CellulosomePlus” consortium (funded by the European Union) that they have successfully tested the use of cellulosomes on a preindustrial scale for bioethanol production from OFMSW, which has helped to increase the technology readiness level (TRL) from TRL 3 to TRL 5.^103^

Synthetic plastics share certain chemical and structural attributes with cellulose, lignin, and cutin as demonstrated by Lee et. al in a review.^104^*Therefore, we hypothesize that incorporating the aforementioned attributes of natural polymer-degrading enzyme systems, such as a multienzyme system with binding domains organized on a scaffold, will facilitate the biological degradation of both hydrolyzable and nonhydrolyzable plastics.* This notion is supported by studies demonstrating the ability of enzymes involved in the degradation of natural polymers to aid in the degradation of PE (i.e., lignin-modifying enzymes) and PET (i.e., cutinases). Consequently, this section outlines the adaptation of anchor modules and multienzyme cascades for the degradation and potential refurbishment of both types of plastics.

### 4.1 Hydrolyzable Polyester Plastic

As discussed previously, complete depolymerization of PET by PETases requires the involvement of multiple enzymes. PHA (polyhydroxyalkanoates) depolymerases, which depolymerize naturally occurring biodegradable polyesters, feature substrate binding modules at their C-terminus, thereby aiding the anchoring of PHA depolymerases to the substrates and facilitating catalysis.^89,105^ Inspired by the strategies employed by PHA depolymerases and cellulosomes, researchers have investigated the integration of adding binding modules as well as using multienzyme scaffolds to optimize the efficient hydrolysis of PET as described below.

#### 4.1.1 Binding Modules

Plastic binding studies have primarily concentrated on re-engineering PET hydrolases by incorporating various binding domains. Many binding domains, including CBMs, chitin binding domains (ChBD), the polyhydroxyalkanoate binding module (PBM), hydrophobins, and peptides such α-SP have been appended to PET hydrolases to improve PET hydrolysis (see Table 2 and references therein). While the literature on PET binding motifs is limited (making it challenging to definitively assess the effects of these modifications on catalytic improvement), the data summarized in Table 2 suggest that the impact of binding domains on activity is more pronounced when utilizing insoluble PET substrates, particularly when the PET hydrolases possess a narrow and deep active site (e.g., cutinases) as opposed to a broad active site (e.g., PETases).

**Table 2:** Improvement in the PET Hydrolase Activities after Fusion to Binding Modules

enzyme	binding module (enzyme and organism source)	substrate	relative fold increase in PET hydrolysis compared to source enzyme
*Thc*Cut1	CBM (cellobiohydrolase I of *Hypocrea jecorina)*	3-PET	1.4^112^
	PBM (cellobiohydrolase I of *Hypocrea jecorina*	3-PET	3.75^112^
*Fs*Cut	CBM-N1 (endoglucanase C of *Cellulomonas fimi)*	pcPET bottles	18^113^
*sThc*Cut1	hydrophobins (HFB4 and HFB7 of *Trichoderma)*	amorphous PET film	16^114^
*Is*PETase^EHA^	CBM (cellobiohydrolase I of *Trichoderma reesei)*	PET granules	1.7^115^
ICCG (mutant of LCC)	α-SP (α-synuclein of *Homo sapiens*)	amorphous PET film	3.89^116^
YCCG (mutant of LCC)	CBMI of *Trichoderma reesi*	2 weight% amorphous PET film	∼4^106^
		>10 weight% amorphous PET film	∼ 1^106^
Tfuc2	dermaseptin SI (*Phyllomedusa*)	PET film	22.7^117^
ICCG (mutant of LCC)	ChBD (*Chitinolyticbacter meiyuanensis* SYBC-H1)	crystalline PET (40%)	11.6^108^
		low-crystalline PET (6.7%)	1.29^108^
	CBM (*Hypocrea jecorina*)	crystalline PET (40%)	7.1^108^
		low-crystalline PET (6.7%)	1.47^108^
*Is*PETase	α-SP (α-synuclein of *Homo sapiens*)	pcPET bottles (crystallinity 32.3%)	3.2–5.1^118^

Though fusing binding modules to PET hydrolases was shown to be promising, it is noteworthy that they do not improve catalytic activity when the solid loading crosses 10 wt % of amorphous PET (Table 2).^106^ PETases have been extensively engineered to degrade amorphous PET,^107^ and it is possible that many can already efficiently bind and access ester bonds in amorphous PET. As a result, the potential impact of binding modules might be more pronounced when dealing with (i) the hydrolysis of highly crystalline plastic materials, as demonstrated by Xue et al. (Table 2),^108^ (ii) processing of mixed plastic materials by directing appended enzymes to their preferred substrate, and (iii) promiscuous enzymes, where binding modules can reduce nonspecific interactions of the enzyme with other substrates.

#### 4.1.2 Improvement in PET Degradation Activity Using Scaffolded Enzymes

The concept of a multienzyme scaffold was applied to the enzymatic hydrolysis of crystalline PET. *Is*PETase, which is particularly effective in the hydrolysis of PET, initially hydrolyzes PET into MHET, which is then further hydrolyzed by MHETase to produce TPA (Figure 1). Alternatively, some lipases or esterases which are capable of efficiently catalyzing the hydrolysis of MHET can be used in conjunction with a PETase for complete hydrolysis as shown in the following report. *Is*PETase and CALB (*Candida antarctica* lipase B) were expressed as fusion proteins linked to the *engE* dockerin of *C. cellulovorans*, and they were subsequently assembled into scaffolding protein CbpA, which contained two corresponding cohesin domains and one CBM3 domain. This resulting 148 kDa complex demonstrated a remarkable 6.5-fold increase in the rate of hydrolysis of crystalline PET (crystallinity 33.9%) compared to a mixture of the individual enzymes. The purpose of utilizing these two PET hydrolyzing enzymes was to achieve an efficient conversion of crystalline PET to TPA.^109^ However, not all multienzyme systems require a scaffold. Often, nature organizes enzymes involved in a multistep process as a large protein complex (direct enzyme–enzyme interactions) or as a single fusion protein (enzymes as domains). This is demonstrated in an independent study where a two-enzyme PETase/MHETase system was explored for efficient PET degradation to TPA by the fusion of an MHETase to a PETase through linker sequences. Fusion proteins were shown to outperform individual enzymes when added at the same concentration.^110^ Finally, while not a fully redesigned scaffold, the application of *Clostridium thermocellum* (naturally expresses cellulosomes) engineered with a thermophilic PET-degrading enzyme (LCC) to degrade PET^111^ is a further example of the potential of the strategy of positioning enzymes in whole-cell applications.

Multienzyme scaffolds can also be applied in plastic upcycling processes. There have been numerous chemobiological upcycling processes reported in the literature aimed at producing value-added compounds such as vanillic acid (perfumes), catechol (perfumes), pyrogallol (sanitizers), muconic acid (polyamides), 2-pyrone-4,6-dicarboxylic acid (polymer materials), polyhydroxyalkanoate (packaging materials), β-ketoadipic acid (engineering plastics), glycolic acid (antioxidants), protocatechuic acid (pharmaceuticals), multifunctional coating materials,^119^ calcium terephthalate (battery anodes),^120^ and *p*-xylylene diamine^121^ (polyamides) as discussed in a couple of excellent reviews.^122,123^ Most of the upcycling processes for PET degradation products involve multiple enzymes for the product synthesis and cofactor regeneration. For instance, the synthesis of *p*-xylylene diamine utilizes carboxylic acid reductases and ω-transaminases for product synthesis, polyphosphate kinase and glucose dehydrogenase for cofactor regeneration, and pyrophosphatase to circumvent enzyme inhibition by pyrophosphate.^121^ The creation of a multienzyme scaffold for such processes may enhance the reusability of enzymes and can potentially increase the rate of synthesis due to proximity effects.

### 4.2 Nonhydrolyzable Plastics

Because of the more recalcitrant C–C backbone found in PS, PE, and PP polymers, it may be especially valuable to consider protein targeting and multienzyme scaffolds as an approach to improve performance. One of the causes of low efficiency of the enzymatic activity on nonhydrolyzable plastics is likely the poor interaction between enzymes and polymer due to inherent structural and chemical differences. Therefore, successful application of some of the features of cellulose-degrading enzyme systems, like substrate binding domains and multienzyme scaffolding complexes, that have been utilized for crystalline PET hydrolysis will also likely prove useful in increasing the rate of enzymatic degradation of nonhydrolyzing plastics.

#### 4.2.1 Binding Modules

Numerous natural and synthetic peptides have been identified that can bind PS and PP, offering a means to target enzymes onto these surfaces.^124^ Random peptide display libraries have been widely used to identify peptides with preferential affinity for a given substrate via rapid screening approaches. This approach has been utilized to identify a number of PS binding peptides, including HWGMWSY,^125^ PS19-6 (RIIIRRIRR),^126^ PS19-6L (RLLLRRLRR),^126^ PS19 (RAFIASRRIKRP),^127^ and PS23 (AGLRLKKAAIHR).^127^ Nonetheless, it is crucial to acknowledge that peptide display libraries can be plagued by the identification of sequences that bind nonspecifically to a wide range of surfaces.^128^ It should also be noted that some of the synthetic peptides identified by these screens have been verified to fall into this category of nonspecific interaction.^129^ In addition, the manner in which the other binding peptides achieve selectivity remains poorly understood. Therefore, before selecting a binding module for plastic degradation, one should perform binding experiments with appropriate controls to ensure that specificity is not compromised, while assessing binding efficiency.

Secretory antimicrobial peptides possess the potential to interact with hydrophobic surfaces in aqueous environments due to their amphiphilic structure. Rübsam et. al identified the PP and PS binding properties of LCI (liquid chromatography peptide I from *Bacillus subtilis*) and TA2 (tachystatin A2 from *Tachypleus tridentatus*) and generated a series of peptides with improved plastic binding affinities by random mutagenesis and directed evolution.^130−132^ These studies therefore provide a pool of plastic-binding peptides that can be screened for fusion with prospective plastic oxidation enzymes to potentially enhance their catalytic activities. Despite these studies on plastic binding antimicrobial peptides (referred to as anchor peptides by the authors), they have not yet been applied to enzymatic plastic degradation.

#### 4.2.2 Cellulosome-Inspired Design of PE Degradation Enzyme Systems

PE and isotactic PP are two of the most challenging plastics to degrade due to their C–C backbone and unreactive C–H groups. Independent linear chains are held together by van der Waals forces to form ordered, closely packed polymers that exhibit high crystallinity. Analogous to cellulose, complete degradation of PE could potentially require synchronized engagement of multiple enzymes, each executing distinct catalytic functions aimed at the breakdown of the polymer to value-added chemicals. This section focuses on enzymes that collectively possess the potential to upcycle PE to alkenes and that could be employed in multienzyme scaffolds.

##### Initial PE-Oxidizing Enzymes

As discussed in detail in a recent review on recycling nonhydrolyzable plastics,^133^ any viable enzymatic processing of PE will likely first require the introduction of oxidized groups into the nonreactive hydrocarbon chains. Despite the importance of applying ligninolytic enzymes like laccase, MnP, and LiP in PE oxidation, these enzymes are excluded from consideration for the proposed multienzyme scaffold in this Perspective due to the lack of experimental evidence on oxidative fragmentation processes using pure enzymes.

The cytochrome P450 superfamily is an exceptional reservoir of potential PE-oxidizing enzymes due to these enzymes promiscuity in oxidizing various organic compounds and their extensive characterization in the literature. For this reason, the cytochromes P450 are considered as possible targets for enzyme engineering to alter their active sites and enhance their ability to oxidize long-chain alkanes.^43^ CYP102A5.v1 from *Bacillus thuringiensis* JNU01 was recently shown to oxidize untreated PE,^44^ although its ability to oxidatively fragment PE into smaller pieces was not reported. Alkane hydroxylases constitute another alternative group of enzymes that could be utilized for the oxidation of PE pretreated to generate linear alkane chains. The availability of alkane hydroxylases with specificity for different carbon atoms in a linear alkane provides a convenient means for upcycling PE into various compounds such as alkanes, alkenes, fatty acids, and diacids tailored to specific applications.^134^

Demetra was recently suggested to be a promising enzyme for the oxidative fragmentation of PE, and it could theoretically be used in a PE upcycling pathway as an initial oxidizing enzyme.^52^ A recent preprint, however, casts doubt on the PE-oxidizing ability of insect hexamerins (which include Demetra), necessitating the confirmation of the PE oxidation activity of Demetra before it can be considered for the pathway.^54^ Another enzyme that has the potential to be used for the upcycling pathway is LMCO2 from *Rhodococcus opacus* R7. This multicopper enzyme was shown to oxidatively breakdown untreated PE powder to produce carboxylic acids (C8–C18) as the primary product.^67^ While information regarding the potential of LMCO2 to oxidize PE blocks (film, sheets, or macro beads) is currently not available, this enzyme could theoretically reduce the number of steps in the upcycling process due to its ability to produce completely oxidized fragmented products, namely carboxylic acids.

##### Example Upcycling Pathway

**Figure 3:**
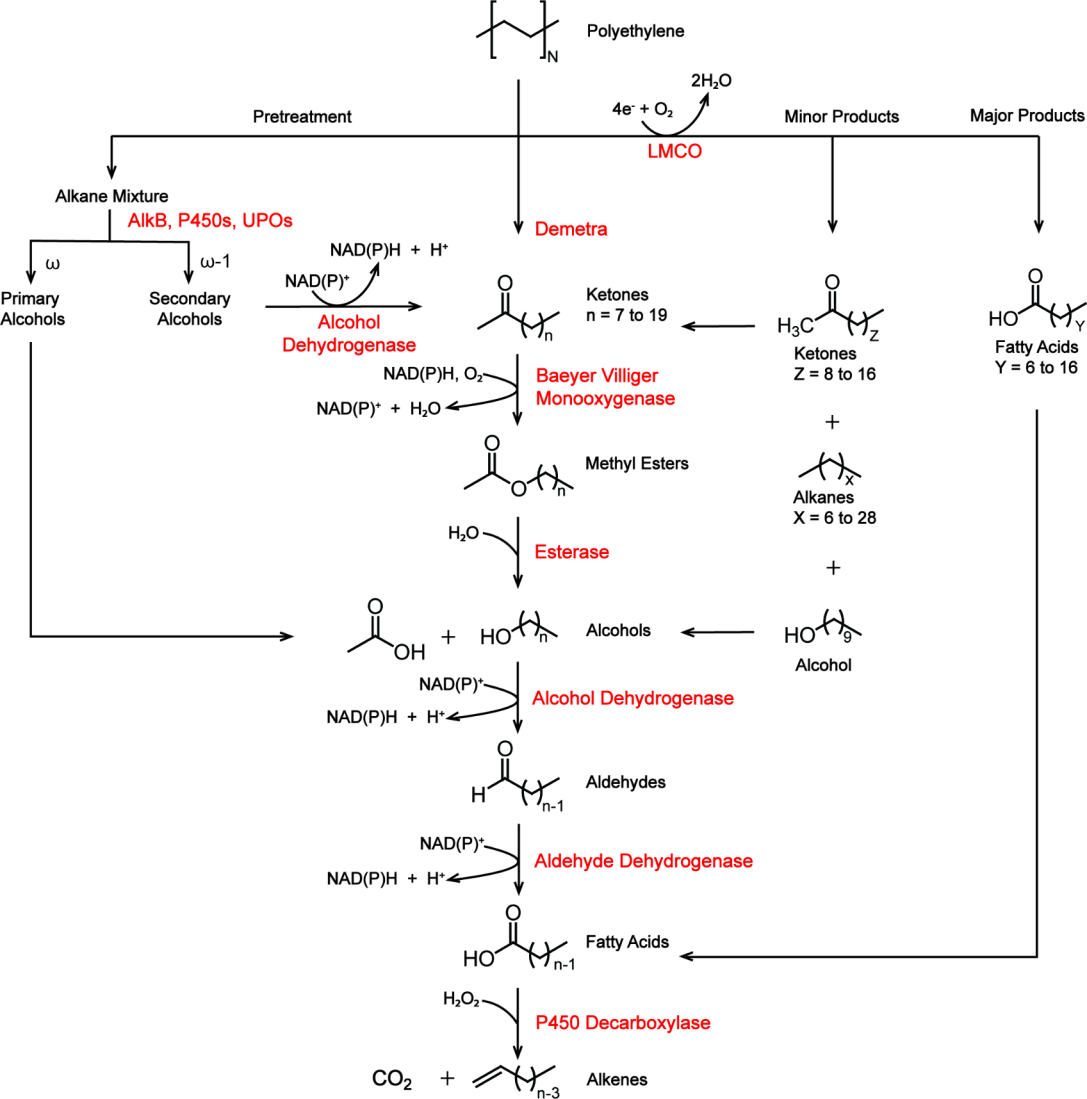
Potential multienzyme pathways for upcycling PE into alkenes: We propose a multienzyme pathway for the efficient upcycling of PE into alkenes. Baeyer–Villiger monooxygenases utilize NAD(P)H as a cofactor to oxidize the ketone mixture (C10–C22) generated by Demetra and produce methyl esters. Cofactor regeneration is accomplished by alcohol dehydrogenase and aldehyde dehydrogenase in downstream reactions, which convert alcohol produced through the hydrolysis of methyl esters into fatty acids. Laccase-like multicopper oxidases (LMCO) can potentially bypass multiple steps in the pathway to directly accumulate fatty acids as the major product. However, the integration of the aforementioned downstream enzymes helps to process any alcohols and ketones (formed as minor products) into the pathway to produce alkenes. Ultimately, a fatty acid decarboxylase, OleTJE (CYP152L1), decarboxylates fatty acids to produce alkenes accompanied by the release of carbon dioxide. Another alternative is the chemoenzymatic pathway wherein chemical methods are employed to form linear chains of alkanes from PE. Alkane hydroxylases, depending on their regiospecificity, produce either primary alcohols or secondary alcohols that can enter the proposed pathway at different points as depicted in the figure to result in the generation of alkenes.

Literature offers numerous types of enzymes that could be used in the subsequent steps of PE upcycling once they are oxidized with or without pretreatment, depending on the desired final product. For example, ketones generated through the oxidative fragmentation of PE by Demetra^52^ or LMCO^67^ can be converted to fatty acid methyl esters (FAME) by a single enzymatic reaction as well as to liquid alkenes through a series of enzymatic reactions (Figure 3). This process begins with the utilization of a flavoprotein known as a Baeyer–Villiger monooxygenase,^43,135,136^ which uses NADH as the electron donor and oxygen as the oxidant to oxidize the ketone mixture into methyl esters.^135,137^ FAMEs, key components of biodiesel,^138^ have various applications as solvents, lubricants, and feedstock chemicals in addition to being used as a fuel.^139^ Subsequently, esterases can be employed to hydrolyze these esters, resulting in a mixture of alcohols and carboxylic acids. In the next steps, alcohols present in the reaction mixture can be oxidized to carboxylic acids with the help of alcohol dehydrogenases and aldehyde dehydrogenases. Zinc-containing alcohol dehydrogenases play a critical role in oxidizing primary alcohols to their respective aldehydes, and aldehyde dehydrogenases can then catalyze the oxidation of aldehydes into carboxylic acids. Importantly, both of these enzymes perform their substrate oxidation by facilitating hydride transfer to NAD^+^,^140,141^ ultimately regenerating NADH for the initial Baeyer–Villiger reaction (as demonstrated by von Haugwitz et al.).^136^ The catalytic decarboxylation of fatty acids (derived from oxidation of aldehydes) by fatty acid decarboxylase produces terminal alkenes that can be used as fuels.^138^ Fatty acid decarboxylase OleTJE (CYP152L1), functioning as a cytochrome P450 peroxygenase, offers a distinct advantage by utilizing a cost-effective cosubstrate, H_2_O_2_, for catalyzing the decarboxylation of fatty acids (C12–C20).^142^

Alternative pathways for upcycling PE, pretreated to generate alkane chains, can be tailor-made to suit our application using a combination of Baeyer–Villiger monooxygenases, alcohol dehydrogenases, aldehyde dehydrogenases, esterases, and decarboxylases. Enzymes typically exhibit specificity for alkanes of a particular chain length (small chain, medium chain, or large chain). Alkane mixtures generated by the pretreatment of PE could, in theory, be selectively oxidized at specific positions (ω, ω-1, within the chain) using dedicated enzymes like alkane hydroxylases, P450s, or peroxygenases to target a product of a particular chain length (Figure 3). Hydroxylation of alkanes with any of these enzymes results in the formation of a primary or secondary alcohols as proposed by Yeom et al.^43^

##### Multienzyme Scaffolds for Upcycling Plastic Breakdown Products

In addition to improving the efficiency of plastic degradation, coordinating multienzyme cascades via an underlying scaffold could also provide benefits for the upcycling of the resulting breakdown products. Indeed, there is now rich literature related to the design and implementation of synthetic scaffolds when it comes to processing soluble intermediates.^143−146^ Briefly, many scaffolding materials, including DNA-based, lipid-based, and protein-based materials, have been extensively explored for the assembly of multienzyme cascades. Interaction domains such as SH3, as well as covalent conjugation tags such as SpyTag, SnoopTag, and HaloTags, have been used to facilitate the assembly of multiple enzymes on these scaffolding materials.^147−149^ The advantages conferred by such rationally designed scaffolding are similar to the advantages observed following compartmentalization and scaffolding in biological systems. For example, coordinating related enzymes with one another in time and space can help: (i) increase pathway flux by substrate channeling/proximity; (ii) reduce the buildup of labile or toxic intermediates; (iii) insulate the desired pathway from competing side reactions while limiting unwanted side-activities of promiscuous enzymes.^146,148,150^

**Figure 4:**
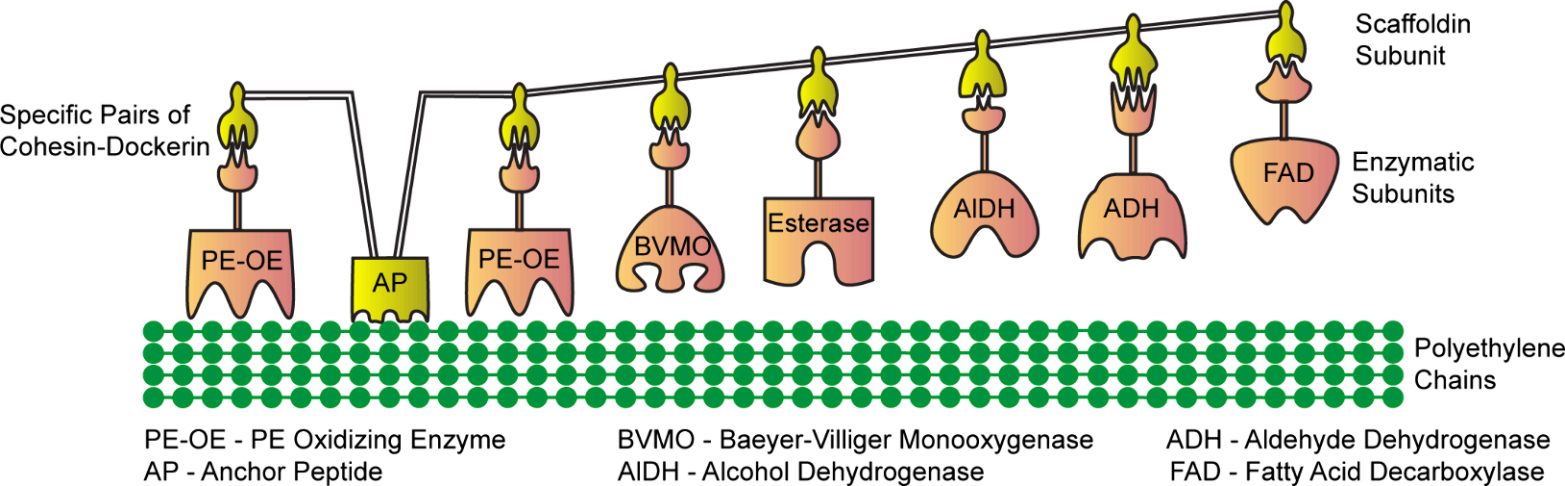
Nature-inspired strategy for the degradation of an inert synthetic plastic: A multienzyme scaffold for PE upcycling is designed in analogy to cellulosome subunits. PE upcycling enzymes can be arranged in a specific order on the scaffold through the design of a scaffold subunit that consists of six different cohesins that interact specifically with a different dockerin. Enzymes are fused to dockerin units to interact with cognate cohesins in scaffoldin, facilitating the desired enzyme order within the scaffold.

Scaffolds inspired by cellulosome properties hold potential advantages in the context of multienzyme cascades for several reasons: (1) Cellulosomes provide a well-studied and proven example whereby coordination of multiple enzymes improves the degradation of analogous recalcitrant natural polymers. (2) The series of cohesin domains in cellulosomes offer multiple binding sites for dockerin bearing catalytic units that can be utilized in a modular fashion. (3) Cohesin–dockerin interactions are species-specific, which provides multiple specific cohesin–dockerin pairs, enabling precise positioning of enzymes on the scaffoldin backbone. This potentially permits the rational engineering of the ordering of enzymes in the series. (4) The assembly of enzymes on scaffoldin can be dynamically controlled by Ca^2+^ concentrations, providing a means to post-translationally regulate enzyme assembly.^151,152^ Discovery of cellulosomes in many new species has facilitated the design of a scaffold with several specific cohesin–dockerin pairs. For example, we provide a potential multienzyme scaffold in Figure 4. It should be noted, however, that large scaffoldin complexes with more than six cohesin units have been difficult to construct thus far.^153^ A recent study published online, while our paper was under revision, successfully displayed a cellulosome-based two-enzyme scaffold on the surface of *E. coli* to degrade PET more efficiently.^154^

In this proof-of-concept scaffoldin design using Demetra/LMCO as the primary oxidizing enzyme, the initial oxidation of untreated and unmodified PE is likely the rate-limiting step, although this can be theoretically improved by fusing a plastic binding anchor peptide to the scaffoldin. Positioning PE-oxidizing enzymes (PE-OE) closer to the binding modules, with one on each side, has the potential to accelerate PE oxidation due to an increased effective local concentration of the enzyme and better substrate anchoring (Figure 4). As the products generated at the end of each catalytic step become increasingly polar, it becomes advantageous to sequentially position the upcycling enzymes farther away from the binding modules, with the final fatty acid decarboxylate enzyme at the far end. In addition to bringing the enzymes involved in the subsequent steps closer together, this configuration offers more flexibility for the upcycling enzymes to access soluble substrates, thereby promoting efficient catalysis. Recently identified enzymes, LMCO2 and LMCO3, exhibit significant promise for incorporation into enzymatic PE upcycling cascades due to their ability to produce fatty acids as the primary oxidized products of PE (Figure 4). This reduces the number of enzymes and cofactors required for the process, effectively reducing the cost. AHs are another group of enzymes that provides versatility in the final products depending on their regiospecificity.^43^ It is crucial to note, however, that there is no literature evidence for the oxidative fragmentation of untreated PE by AHs. Thus, it is important to use pretreatments to produce linear chains of alkanes before the use of AHs in the pathway.

One of the major limitations of multienzyme processes involving oxidoreductive reactions is the requirement of expensive cofactors. An ideal multienzyme reaction would continuously generate and reuse cofactors within the system. When this is not possible, additional enzymes with cheaper substrates, such as alcohol dehydrogenase, formate dehydrogenase, or glucose dehydrogenase, should be included in the multienzyme scaffold for cofactor regeneration.^155^ For example, the proposed sequential reactions consume two molecules of NAD(P)^+^ (by alcohol and aldehyde dehydrogenases) but regenerate only one NAD(P)^+^ (by BVMO). Thus, this pathway would benefit from a cofactor regeneration system. Depending on the number and type of cofactors required for the upcycling pathway, optimization of the cofactor regeneration system is extremely important for multienzyme cascades.

Another factor to consider when designing a multienzyme cascade is that changing reaction conditions can differentially impact the kinetics and stability of the participating enzymes, especially when those enzymes are from different classes or pathways. Kinetic modeling of multienzyme processes that takes into account the properties of all participating enzymes would simplify the complexity of the process design and help ensure a reliable process when operating conditions vary.^156^ Additionally, some scaffolding designs can actually diminish overall enzyme activity, for example, by creating a steric clash between the active site and the positioning of the bound polymer substrate.^157^ Any scaffolding approach will need to address such limitations, possibly by replicating properties of native linkers from cellulosomal enzymes that provide the length and flexibility needed to give bound enzymes appropriate access to the substrate.^158^

## 5 Concluding Remarks

This Perspective suggests new avenues for advancing enzymatic plastic degradation by exploring cellulosome-inspired strategies. Synthetic plastics are relatively new to the environment, and nature has not yet had the opportunity to develop an optimized plastic degradation process. Nature’s optimized degradation pathways for natural polymers such as cellulose provide a promising blueprint for the development of efficient plastic degradation technologies. The successful enhancement of crystalline PET hydrolysis through the addition of a binding module, along with the remarkable ability of cellulases to efficiently degrade crystalline cellulose when the enzymes are linked on a cellulosome scaffold, serves as the driving force behind the proposed strategy for upcycling PE. In the design of multienzyme scaffolds for plastic upcycling processes, it is imperative to carefully consider the characteristics of enzymes (stability, plastic binding affinity, active site shape and structure, etc.) and specific attributes of the postconsumer material (homogeneity, crystallinity, additives composition, and contaminants). Fortuitously, linking enzymes to a scaffold could also aid in the recovery and reuse of the enzymes from batch to batch. Finally, scaffolding multiple enzymes that are part of the same pathway can theoretically improve the efficiency of the enzymatic cascade, thereby enabling a viable and sustainable solution to plastic pollution.

Although modifying cellulosomes and related natural scaffolding strategies may be an effective approach for improving the biological breakdown of difficult substrates, it is important to acknowledge that this does not necessarily mean that it will readily translate to an industrial process. The literature on plastic-degrading enzymes and processes is rapidly developing, and it is difficult to anticipate the details of future scaled industrial processes for plastic breakdown/upcycling. Nonetheless, reference to the use of analogous scaled approaches for the breakdown of lignocellulosic materials at scale may provide a useful comparison.^103^ Taken together, we propose that this nature-inspired approach is valuable to consider in the development of new processes for the degradation and subsequent upcycling of plastic.

## References

[ref1] Bioplastic Industry Worldwide; Statista, 2023.

[ref2] Mohanan N.; Montazer Z.; Sharma P. K.; Levin D. B. Microbial and Enzymatic Degradation of Synthetic Plastics. Front. Microbiol. 2020, 11, 580709 10.3389/fmicb.2020.580709. 33324366 PMC7726165

[ref3] PlasticsEurope. Plastics - the Facts 2022. An Analysis of European Plastics Production, Demand, Conversion and End-of-Life Management; Plastics Europe: Belgium, 2022.

[ref4] Rujnić-Sokele M.; Pilipović A. Challenges and Opportunities of Biodegradable Plastics: A Mini Review. Waste Manag Res. 2017, 35 (2), 132–140. 10.1177/0734242X16683272. 28064843

[ref5] Shen M.; Song B.; Zeng G.; Zhang Y.; Huang W.; Wen X.; Tang W. Are Biodegradable Plastics a Promising Solution to Solve the Global Plastic Pollution?. Environ. Pollut. 2020, 263, 114469 10.1016/j.envpol.2020.114469. 32272422

[ref6] Schyns Z. O. G.; Shaver M. P. Mechanical Recycling of Packaging Plastics: A Review. Macromol. Rapid Commun. 2021, 42 (3), 2000415 10.1002/marc.202000415. 33000883

[ref7] Huang J.; Veksha A.; Chan W. P.; Giannis A.; Lisak G. Chemical Recycling of Plastic Waste for Sustainable Material Management: A Prospective Review on Catalysts and Processes. Renewable and Sustainable Energy Reviews 2022, 154, 111866 10.1016/j.rser.2021.111866.

[ref8] Mitra B.; Das A. Microbes and Environment Sustainability: An in-Depth Review on the Role of Insect Gut Microbiota in Plastic Biodegradation. Synergistic Approaches for Bioremediation of Environmental Pollutants: Recent Advances and Challenges; Elsevier, 2022; pp 1–25. 10.1016/B978-0-323-91860-2.00013-0.

[ref9] Gilani I. E.; Sayadi S.; Zouari N.; Al-Ghouti M. A. Plastic Waste Impact and Biotechnology: Exploring Polymer Degradation, Microbial Role, and Sustainable Development Implications. Bioresource Technology Reports 2023, 24, 101606 10.1016/j.biteb.2023.101606.

[ref10] Mitra B.; Das A. The Ability of Insects to Degrade Complex Synthetic Polymers. Arthropods - New Advances and Perspectives; IntechOpen, 2023; p 106948. 10.5772/intechopen.106948.

[ref11] Seo M.-J.; Yun S.-D.; Kim H.-W.; Yeom S.-J. Polyethylene-Biodegrading Microbes and Their Future Directions. Biotechnol Bioproc E 2023, 28 (6), 977–989. 10.1007/s12257-022-0264-9.

[ref12] Cai Z.; Li M.; Zhu Z.; Wang X.; Huang Y.; Li T.; Gong H.; Yan M. Biological Degradation of Plastics and Microplastics: A Recent Perspective on Associated Mechanisms and Influencing Factors. Microorganisms 2023, 11 (7), 1661 10.3390/microorganisms11071661. 37512834 PMC10386651

[ref13] Tournier V.; Duquesne S.; Guillamot F.; Cramail H.; Taton D.; Marty A.; André I. Enzymes’ Power for Plastics Degradation. Chem. Rev. 2023, 123 (9), 5612–5701. 10.1021/acs.chemrev.2c00644. 36916764

[ref14] Khairul Anuar N. F. S.; Huyop F.; Ur-Rehman G.; Abdullah F.; Normi Y. M.; Sabullah M. K.; Abdul Wahab R. An Overview into Polyethylene Terephthalate (PET) Hydrolases and Efforts in Tailoring Enzymes for Improved Plastic Degradation. IJMS 2022, 23 (20), 12644 10.3390/ijms232012644. 36293501 PMC9603852

[ref15] Han Y.; Wang R.; Wang D.; Luan Y. Enzymatic Degradation of Synthetic Plastics by Hydrolases/Oxidoreductases. International Biodeterioration & Biodegradation 2024, 189, 105746 10.1016/j.ibiod.2024.105746.

[ref16] Shi L.; Zhu L. Recent Advances and Challenges in Enzymatic Depolymerization and Recycling of PET Wastes. ChemBioChem. 2024, 25 (2), e202300578 10.1002/cbic.202300578. 37960968

[ref17] Zheng L.; Wang M.; Li Y.; Xiong Y.; Wu C. Recycling and Degradation of Polyamides. Molecules 2024, 29 (8), 1742 10.3390/molecules29081742. 38675560 PMC11052090

[ref18] Liu J.; He J.; Xue R.; Xu B.; Qian X.; Xin F.; Blank L. M.; Zhou J.; Wei R.; Dong W.; Jiang M. Biodegradation and Up-Cycling of Polyurethanes: Progress, Challenges, and Prospects. Biotechnology Advances 2021, 48, 107730 10.1016/j.biotechadv.2021.107730. 33713745

[ref19] Novy V.; Carneiro L. V.; Shin J. H.; Larsbrink J.; Olsson L. Phylogenetic Analysis and In-Depth Characterization of Functionally and Structurally Diverse CE5 Cutinases. J. Biol. Chem. 2021, 297 (5), 101302 10.1016/j.jbc.2021.101302. 34653507 PMC8577158

[ref20] Kawai F.; Kawabata T.; Oda M. Current Knowledge on Enzymatic PET Degradation and Its Possible Application to Waste Stream Management and Other Fields. Appl. Microbiol. Biotechnol. 2019, 103 (11), 4253–4268. 10.1007/s00253-019-09717-y. 30957199 PMC6505623

[ref21] Müller R.-J.; Schrader H.; Profe J.; Dresler K.; Deckwer W.-D. Enzymatic Degradation of Poly(Ethylene Terephthalate): Rapid Hydrolyse Using a Hydrolase fromT. Fusca. Macromol. Rapid Commun. 2005, 26 (17), 1400–1405. 10.1002/marc.200500410.

[ref22] Then J.; Wei R.; Oeser T.; Barth M.; Belisário-Ferrari M. R.; Schmidt J.; Zimmermann W. Ca ^2+^ and Mg ^2+^ Binding Site Engineering Increases the Degradation of Polyethylene Terephthalate Films by Polyester Hydrolases from *Thermobifida Fusca*. Biotechnology Journal 2015, 10 (4), 592–598. 10.1002/biot.201400620. 25545638

[ref23] Herrero Acero E.; Ribitsch D.; Steinkellner G.; Gruber K.; Greimel K.; Eiteljoerg I.; Trotscha E.; Wei R.; Zimmermann W.; Zinn M.; Cavaco-Paulo A.; Freddi G.; Schwab H.; Guebitz G. Enzymatic Surface Hydrolysis of PET: Effect of Structural Diversity on Kinetic Properties of Cutinases from Thermobifida. Macromolecules 2011, 44 (12), 4632–4640. 10.1021/ma200949p.

[ref24] Ribitsch D.; Acero E. H.; Greimel K.; Eiteljoerg I.; Trotscha E.; Freddi G.; Schwab H.; Guebitz G. M. Characterization of a New Cutinase from *Thermobifida Alba* for PET-Surface Hydrolysis. Biocatalysis and Biotransformation 2012, 30 (1), 2–9. 10.3109/10242422.2012.644435.

[ref25] Sulaiman S.; Yamato S.; Kanaya E.; Kim J.-J.; Koga Y.; Takano K.; Kanaya S. Isolation of a Novel Cutinase Homolog with Polyethylene Terephthalate-Degrading Activity from Leaf-Branch Compost by Using a Metagenomic Approach. Appl. Environ. Microbiol. 2012, 78 (5), 1556–1562. 10.1128/AEM.06725-11. 22194294 PMC3294458

[ref26] Fritzsche S.; Tischer F.; Peukert W.; Castiglione K. You Get What You Screen for: A Benchmark Analysis of Leaf Branch Compost Cutinase Variants for Polyethylene Terephthalate (PET) Degradation. React. Chem. Eng. 2023, 8, 2156–2169. 10.1039/D3RE00056G.

[ref27] Shirke A. N.; White C.; Englaender J. A.; Zwarycz A.; Butterfoss G. L.; Linhardt R. J.; Gross R. A. Stabilizing Leaf and Branch Compost Cutinase (LCC) with Glycosylation: Mechanism and Effect on PET Hydrolysis. Biochemistry 2018, 57 (7), 1190–1200. 10.1021/acs.biochem.7b01189. 29328676

[ref28] Andlar M.; Rezić T.; Marđetko N.; Kracher D.; Ludwig R.; Šantek B. Lignocellulose Degradation: An Overview of Fungi and Fungal Enzymes Involved in Lignocellulose Degradation. Eng. Life Sci. 2018, 18 (11), 768–778. 10.1002/elsc.201800039. 32624871 PMC6999254

[ref29] Hu X.; Thumarat U.; Zhang X.; Tang M.; Kawai F. Diversity of Polyester-Degrading Bacteria in Compost and Molecular Analysis of a Thermoactive Esterase from Thermobifida Alba AHK119. Appl. Microbiol. Biotechnol. 2010, 87 (2), 771–779. 10.1007/s00253-010-2555-x. 20393707

[ref30] Ribitsch D.; Herrero Acero E.; Greimel K.; Dellacher A.; Zitzenbacher S.; Marold A.; Rodriguez R. D.; Steinkellner G.; Gruber K.; Schwab H.; Guebitz G. M. A New Esterase from Thermobifida Halotolerans Hydrolyses Polyethylene Terephthalate (PET) and Polylactic Acid (PLA). Polymers 2012, 4 (1), 617–629. 10.3390/polym4010617.

[ref31] Carr C. M.; Clarke D. J.; Dobson A. D. W. Microbial Polyethylene Terephthalate Hydrolases: Current and Future Perspectives. Front. Microbiol. 2020, 11, 571265 10.3389/fmicb.2020.571265. 33262744 PMC7686037

[ref32] Kawai F. The Current State of Research on PET Hydrolyzing Enzymes Available for Biorecycling. Catalysts 2021, 11 (2), 206 10.3390/catal11020206.

[ref33] Yoshida S.; Hiraga K.; Takehana T.; Taniguchi I.; Yamaji H.; Maeda Y.; Toyohara K.; Miyamoto K.; Kimura Y.; Oda K. A Bacterium That Degrades and Assimilates Poly(Ethylene Terephthalate). Science 2016, 351 (6278), 1196–1199. 10.1126/science.aad6359. 26965627

[ref34] Austin H. P.; Allen M. D.; Donohoe B. S.; Rorrer N. A.; Kearns F. L.; Silveira R. L.; Pollard B. C.; Dominick G.; Duman R.; El Omari K.; Mykhaylyk V.; Wagner A.; Michener W. E.; Amore A.; Skaf M. S.; Crowley M. F.; Thorne A. W.; Johnson C. W.; Woodcock H. L.; McGeehan J. E.; Beckham G. T. Characterization and Engineering of a Plastic-Degrading Aromatic Polyesterase. Proc. Natl. Acad. Sci. U.S.A. 2018, 115 (19), E4350-E4357 10.1073/pnas.1718804115. 29666242 PMC5948967

[ref35] Berselli A.; Ramos M. J.; Menziani M. C. Novel Pet-Degrading Enzymes: Structure-Function from a Computational Perspective. ChemBioChem. 2021, 22 (12), 2032–2050. 10.1002/cbic.202000841. 33470503

[ref36] Alam I.; Aalismail N.; Martin C.; Kamau A.; Guzmán-Vega F. J.; Jamil T.; Momin A. A.; Acinas S. G.; Gasol J. M.; Arold S. T.; Gojobori T.; Agusti S.; Duarte C. M. Rapid Evolution of Plastic-Degrading Enzymes Prevalent in the Global Ocean; preprint; Evolutionary Biology, 2020; p 2020. 10.1101/2020.09.07.285692.

[ref37] Joho Y.; Vongsouthi V.; Spence M. A.; Ton J.; Gomez C.; Tan L. L.; Kaczmarski J. A.; Caputo A. T.; Royan S.; Jackson C. J.; Ardevol A. Ancestral Sequence Reconstruction Identifies Structural Changes Underlying the Evolution of *Ideonella Sakaiensis* PETase and Variants with Improved Stability and Activity. Biochemistry 2023, 62 (2), 437–450. 10.1021/acs.biochem.2c00323. 35951410

[ref38] Sonnendecker C.; Oeser J.; Richter P. K.; Hille P.; Zhao Z.; Fischer C.; Lippold H.; Blázquez-Sánchez P.; Engelberger F.; Ramírez-Sarmiento C. A.; Oeser T.; Lihanova Y.; Frank R.; Jahnke H.; Billig S.; Abel B.; Sträter N.; Matysik J.; Zimmermann W. Low Carbon Footprint Recycling of Post-Consumer PET Plastic with a Metagenomic Polyester Hydrolase. ChemSusChem 2022, 15 (9), e202101062 10.1002/cssc.202101062. 34129279 PMC9303343

[ref39] Bollinger A.; Thies S.; Knieps-Grünhagen E.; Gertzen C.; Kobus S.; Höppner A.; Ferrer M.; Gohlke H.; Smits S. H. J.; Jaeger K.-E. A Novel Polyester Hydrolase From the Marine Bacterium Pseudomonas Aestusnigri - Structural and Functional Insights. Front. Microbiol. 2020, 11, 114 10.3389/fmicb.2020.00114. 32117139 PMC7031157

[ref40] Zhang Y.; Pedersen J. N.; Eser B. E.; Guo Z. Biodegradation of Polyethylene and Polystyrene: From Microbial Deterioration to Enzyme Discovery. Biotechnology Advances 2022, 60, 107991 10.1016/j.biotechadv.2022.107991. 35654281

[ref41] Rana A. K.; Thakur M. K.; Saini A. K.; Mokhta S. K.; Moradi O.; Rydzkowski T.; Alsanie W. F.; Wang Q.; Grammatikos S.; Thakur V. K. Recent Developments in Microbial Degradation of Polypropylene: Integrated Approaches towards a Sustainable Environment. Science of The Total Environment 2022, 826, 154056 10.1016/j.scitotenv.2022.154056. 35231525

[ref42] Cowan A. R.; Costanzo C. M.; Benham R.; Loveridge E. J.; Moody S. C. Fungal Bioremediation of Polyethylene: Challenges and Perspectives. J. Appl. Microbiol. 2022, 132 (1), 78–89. 10.1111/jam.15203. 34218487

[ref43] Yeom S.-J.; Le T.-K.; Yun C.-H. P450-Driven Plastic-Degrading Synthetic Bacteria. Trends Biotechnol. 2022, 40 (2), 166–179. 10.1016/j.tibtech.2021.06.003. 34243985

[ref44] Yun S.-D.; Lee C. O.; Kim H.-W.; An S. J.; Kim S.; Seo M.-J.; Park C.; Yun C.-H.; Chi W. S.; Yeom S.-J. Exploring a New Biocatalyst from *Bacillus Thuringiensis* JNU01 for Polyethylene Biodegradation. Environ. Sci. Technol. Lett. 2023, 10 (6), 485–492. 10.1021/acs.estlett.3c00189.

[ref45] Ji Y.; Mao G.; Wang Y.; Bartlam M. Structural Insights into Diversity and N-Alkane Biodegradation Mechanisms of Alkane Hydroxylases. Front. Microbiol. 2013, 4, 42005 10.3389/fmicb.2013.00058. PMC3604635 23519435

[ref46] Chunyan X.; Qaria M. A.; Qi X.; Daochen Z. The Role of Microorganisms in Petroleum Degradation: Current Development and Prospects. Science of The Total Environment 2023, 865, 161112 10.1016/j.scitotenv.2022.161112. 36586680

[ref47] Jeon H. J.; Kim M. N. Comparison of the Functional Characterization between Alkane Monooxygenases for Low-Molecular-Weight Polyethylene Biodegradation. International Biodeterioration & Biodegradation 2016, 114, 202–208. 10.1016/j.ibiod.2016.06.012.

[ref48] Gyung Yoon M.; Jeong Jeon H.; Nam Kim M. Biodegradation of Polyethylene by a Soil Bacterium and AlkB Cloned Recombinant Cell. J. Bioremed Biodegrad 2012, 03 (04), 1–8. 10.4172/2155-6199.1000145.

[ref49] Tan Q.; Chen W.; Liu H.; Yan W.; Huang X.; Li Y. The Programmed Sequence-Based Oxygenase Screening for Polypropylene Degradation. Journal of Hazardous Materials 2024, 465, 133173 10.1016/j.jhazmat.2023.133173. 38061126

[ref50] Zhang Z.; Peng H.; Yang D.; Zhang G.; Zhang J.; Ju F. Polyvinyl Chloride Degradation by a Bacterium Isolated from the Gut of Insect Larvae. Nat. Commun. 2022, 13 (1), 5360 10.1038/s41467-022-32903-y. 36097154 PMC9468159

[ref51] Cassone B. J.; Grove H. C.; Elebute O.; Villanueva S. M. P.; LeMoine C. M. R. Role of the Intestinal Microbiome in Low-Density Polyethylene Degradation by Caterpillar Larvae of the Greater Wax Moth, *Galleria Mellonella*. Proc. R. Soc. B 2020, 287 (1922), 20200112 10.1098/rspb.2020.0112. PMC7126078 32126962

[ref52] Sanluis-Verdes A.; Colomer-Vidal P.; Rodriguez-Ventura F.; Bello-Villarino M.; Spinola-Amilibia M.; Ruiz-Lopez E.; Illanes-Vicioso R.; Castroviejo P.; Aiese Cigliano R.; Montoya M.; Falabella P.; Pesquera C.; Gonzalez-Legarreta L.; Arias-Palomo E.; Solà M.; Torroba T.; Arias C. F.; Bertocchini F. Wax Worm Saliva and the Enzymes Therein Are the Key to Polyethylene Degradation by Galleria Mellonella. Nat. Commun. 2022, 13 (1), 5568 10.1038/s41467-022-33127-w. 36195604 PMC9532405

[ref53] Spínola-Amilibia M.; Illanes-Vicioso R.; Ruiz-Lopez E.; Colomer-Vidal P.; Rodriguez-Ventura F. V.; Peces Pérez R.; Arias C. F.; Torroba T.; Solá M.; Arias-Palomo E.; Bertocchini F. Plastic Degradation by Insect Hexamerins: Near-Atomic Resolution Structures of the Polyethylene-Degrading Proteins from the Wax Worm Saliva. Sci. Adv. 2023, 9 (38), eadi6813 10.1126/sciadv.adi6813. 37729416 PMC10511194

[ref54] Stepnov A. A.; Lopez-Tavera E.; Klauer R.; Lincoln C. L.; Chowreddy R. R.; Beckham G. T.; Eijsink V. G. H.; Solomon K.; Blenner M.; Vaaje-Kolstad G. Revisiting the Activity of Two Poly(Vinyl Chloride)- and Polyethylene-Degrading Enzymes. bioRxiv March 15, 2024. 10.1101/2024.03.15.585159. PMC11445424 39353919

[ref55] Lear G.; Maday S. D. M.; Gambarini V.; Northcott G.; Abbel R.; Kingsbury J. M.; Weaver L.; Wallbank J. A.; Pantos O. Microbial Abilities to Degrade Global Environmental Plastic Polymer Waste Are Overstated. Environ. Res. Lett. 2022, 17 (4), 043002 10.1088/1748-9326/ac59a7.

[ref56] Kumar A.; Chandra R. Ligninolytic Enzymes and Its Mechanisms for Degradation of Lignocellulosic Waste in Environment. Heliyon 2020, 6 (2), e03170 10.1016/j.heliyon.2020.e03170. 32095645 PMC7033530

[ref57] Reiss R.; Ihssen J.; Richter M.; Eichhorn E.; Schilling B.; Thöny-Meyer L. Laccase versus Laccase-Like Multi-Copper Oxidase: A Comparative Study of Similar Enzymes with Diverse Substrate Spectra. PLoS One 2013, 8 (6), e65633 10.1371/journal.pone.0065633. 23755261 PMC3670849

[ref58] Jones S. M.; Solomon E. I. Electron Transfer and Reaction Mechanism of Laccases. Cell. Mol. Life Sci. 2015, 72 (5), 869–883. 10.1007/s00018-014-1826-6. 25572295 PMC4323859

[ref59] Yao C.; Xia W.; Dou M.; Du Y.; Wu J. Oxidative Degradation of UV-Irradiated Polyethylene by Laccase-Mediator System. Journal of Hazardous Materials 2022, 440, 129709 10.1016/j.jhazmat.2022.129709. 35939906

[ref60] Fujisawa M.; Hirai H.; Nishida T. Degradation of Polyethylene and Nylon-66 by the Laccase-Mediator System. Journal of Polymers and the Environment 2001, 9 (3), 103–108. 10.1023/A:1020472426516.

[ref61] Sheik S.; Chandrashekar K. R.; Swaroop K.; Somashekarappa H. M. Biodegradation of Gamma Irradiated Low Density Polyethylene and Polypropylene by Endophytic Fungi. International Biodeterioration & Biodegradation 2015, 105, 21–29. 10.1016/j.ibiod.2015.08.006.

[ref62] Hock O. G.; Lum H. W.; Qin D. D.; Kee W. K.; Shing W. L. The Growth and Laccase Activity of Edible Mushrooms Involved in Plastics Degradation. Toxicology 2019, 15, 57.

[ref63] Zhang J.; Gao D.; Li Q.; Zhao Y.; Li L.; Lin H.; Bi Q.; Zhao Y. Biodegradation of Polyethylene Microplastic Particles by the Fungus Aspergillus Flavus from the Guts of Wax Moth Galleria Mellonella. Science of The Total Environment 2020, 704, 135931 10.1016/j.scitotenv.2019.135931. 31830656

[ref64] Sowmya H. V.; Ramalingappa B.; Nayanashree G.; Thippeswamy B.; Krishnappa M. Polyethylene Degradation by Fungal Consortium. International Journal of Environmental Research 2015, 9 (3), 823–830.

[ref65] Santo M.; Weitsman R.; Sivan A. The Role of the Copper-Binding Enzyme - Laccase - in the Biodegradation of Polyethylene by the Actinomycete Rhodococcus Ruber. International Biodeterioration & Biodegradation 2013, 84, 204–210. 10.1016/j.ibiod.2012.03.001.

[ref66] Zhang Y.; Plesner T. J.; Ouyang Y.; Zheng Y.-C.; Bouhier E.; Berentzen E. I.; Zhang M.; Zhou P.; Zimmermann W.; Andersen G. R.; Eser B. E.; Guo Z. Computer-Aided Discovery of a Novel Thermophilic Laccase for Low-Density Polyethylene Degradation. Journal of Hazardous Materials 2023, 458, 131986 10.1016/j.jhazmat.2023.131986. 37413797

[ref67] Zampolli J.; Mangiagalli M.; Vezzini D.; Lasagni M.; Ami D.; Natalello A.; Arrigoni F.; Bertini L.; Lotti M.; Di Gennaro P. Oxidative Degradation of Polyethylene by Two Novel Laccase-like Multicopper Oxidases from Rhodococcus Opacus R7. Environmental Technology & Innovation 2023, 32, 103273 10.1016/j.eti.2023.103273.

[ref68] Nakamiya K.; Sakasita G.; Ooi T.; Kinoshita S. Enzymatic Degradation of Polystyrene by Hydroquinone Peroxidase of Azotobacter Beijerinckii HM121. Journal of Fermentation and Bioengineering 1997, 84 (5), 480–482. 10.1016/S0922-338X(97)82013-2.

[ref69] Mahor D.; Cong Z.; Weissenborn M. J.; Hollmann F.; Zhang W. Valorization of Small Alkanes by Biocatalytic Oxyfunctionalization. ChemSusChem 2022, 15 (9), e202101116 10.1002/cssc.202101116. 34288540

[ref70] Bar-Even A.; Noor E.; Savir Y.; Liebermeister W.; Davidi D.; Tawfik D. S.; Milo R. The Moderately Efficient Enzyme: Evolutionary and Physicochemical Trends Shaping Enzyme Parameters. Biochemistry 2011, 50 (21), 4402–4410. 10.1021/bi2002289. 21506553

[ref71] Son H. F.; Cho I. J.; Joo S.; Seo H.; Sagong H.-Y.; Choi S. Y.; Lee S. Y.; Kim K.-J. Rational Protein Engineering of Thermo-Stable PETase from *Ideonella Sakaiensis* for Highly Efficient PET Degradation. ACS Catal. 2019, 9 (4), 3519–3526. 10.1021/acscatal.9b00568.

[ref72] Lu H.; Diaz D. J.; Czarnecki N. J.; Zhu C.; Kim W.; Shroff R.; Acosta D. J.; Alexander B. R.; Cole H. O.; Zhang Y.; Lynd N. A.; Ellington A. D.; Alper H. S. Machine Learning-Aided Engineering of Hydrolases for PET Depolymerization. Nature 2022, 604 (7907), 662–667. 10.1038/s41586-022-04599-z. 35478237

[ref73] Bell E. L.; Smithson R.; Kilbride S.; Foster J.; Hardy F. J.; Ramachandran S.; Tedstone A. A.; Haigh S. J.; Garforth A. A.; Day P. J. R.; Levy C.; Shaver M. P.; Green A. P. Directed Evolution of an Efficient and Thermostable PET Depolymerase. Nat. Catal 2022, 5 (8), 673–681. 10.1038/s41929-022-00821-3.

[ref74] Cui Y.; Chen Y.; Liu X.; Dong S.; Tian Y.; Qiao Y.; Mitra R.; Han J.; Li C.; Han X.; Liu W.; Chen Q.; Wei W.; Wang X.; Du W.; Tang S.; Xiang H.; Liu H.; Liang Y.; Houk K. N.; Wu B. Computational Redesign of a PETase for Plastic Biodegradation under Ambient Condition by the GRAPE Strategy. ACS Catal. 2021, 11 (3), 1340–1350. 10.1021/acscatal.0c05126.

[ref75] Shi L.; Liu P.; Tan Z.; Zhao W.; Gao J.; Gu Q.; Ma H.; Liu H.; Zhu L. Complete Depolymerization of PET Wastes by an Evolved PET Hydrolase from Directed Evolution. Angew. Chem. Int. Ed 2023, 62 (14), e202218390 10.1002/anie.202218390. 36751696

[ref76] Wu B.; Cui Y.; Chen Y.; Sun J.; Zhu T.; Pang H.; Li C.; Geng W. Deep Learning-Aided Redesign of a Hydrolase for near 100% PET Depolymerization under Industrially Relevant Conditions.. Research Square 2023, preprint; in review 10.21203/rs.3.rs-2465520/v1.

[ref77] Tournier V.; Topham C. M.; Gilles A.; David B.; Folgoas C.; Moya-Leclair E.; Kamionka E.; Desrousseaux M.-L.; Texier H.; Gavalda S.; Cot M.; Guémard E.; Dalibey M.; Nomme J.; Cioci G.; Barbe S.; Chateau M.; André I.; Duquesne S.; Marty A. An Engineered PET Depolymerase to Break down and Recycle Plastic Bottles. Nature 2020, 580, 216–219. 10.1038/s41586-020-2149-4. 32269349

[ref78] Pfaff L.; Gao J.; Li Z.; Jäckering A.; Weber G.; Mican J.; Chen Y.; Dong W.; Han X.; Feiler C. G.; Ao Y.-F.; Badenhorst C. P. S.; Bednar D.; Palm G. J.; Lammers M.; Damborsky J.; Strodel B.; Liu W.; Bornscheuer U. T.; Wei R. Multiple Substrate Binding Mode-Guided Engineering of a Thermophilic PET Hydrolase. ACS Catal. 2022, 12 (15), 9790–9800. 10.1021/acscatal.2c02275. 35966606 PMC9361285

[ref79] Li W. F.; Zhou X. X.; Lu P. Structural Features of Thermozymes. Biotechnology Advances 2005, 23 (4), 271–281. 10.1016/j.biotechadv.2005.01.002. 15848038

[ref80] Roth C.; Wei R.; Oeser T.; Then J.; Föllner C.; Zimmermann W.; Sträter N. Structural and Functional Studies on a Thermostable Polyethylene Terephthalate Degrading Hydrolase from Thermobifida Fusca. Appl. Microbiol. Biotechnol. 2014, 98 (18), 7815–7823. 10.1007/s00253-014-5672-0. 24728714

[ref81] Pirillo V.; Orlando M.; Battaglia C.; Pollegioni L.; Molla G. Efficient Polyethylene Terephthalate Degradation at Moderate Temperature: A Protein Engineering Study of LC -cutinase Highlights the Key Role of Residue 243. FEBS Journal 2023, 290 (12), 3185–3202. 10.1111/febs.16736. 36695006

[ref82] Wei R.; Oeser T.; Schmidt J.; Meier R.; Barth M.; Then J.; Zimmermann W. Engineered Bacterial Polyester Hydrolases Efficiently Degrade Polyethylene Terephthalate Due to Relieved Product Inhibition: Engineered Polyester Hydrolases. Biotechnol. Bioeng. 2016, 113 (8), 1658–1665. 10.1002/bit.25941. 26804057

[ref83] Zadjelovic V.; Erni-Cassola G.; Obrador-Viel T.; Lester D.; Eley Y.; Gibson M. I.; Dorador C.; Golyshin P. N.; Black S.; Wellington E. M. H.; Christie-Oleza J. A. A Mechanistic Understanding of Polyethylene Biodegradation by the Marine Bacterium Alcanivorax. Journal of Hazardous Materials 2022, 436, 129278 10.1016/j.jhazmat.2022.129278. 35739790

[ref84] Silva C.; Da S.; Silva N.; Matamá T.; Araújo R.; Martins M.; Chen S.; Chen J.; Wu J.; Casal M.; Cavaco-Paulo A. De Biotechnology Journal 2011, 6 (10), 1230–1239. 10.1002/biot.201000391. 21751386

[ref85] Chen K.; Hu Y.; Dong X.; Sun Y. Molecular Insights into the Enhanced Performance of EKylated PETase Toward PET Degradation. ACS Catal. 2021, 11 (12), 7358–7370. 10.1021/acscatal.1c01062.

[ref86] Barth M.; Oeser T.; Wei R.; Then J.; Schmidt J.; Zimmermann W. Effect of Hydrolysis Products on the Enzymatic Degradation of Polyethylene Terephthalate Nanoparticles by a Polyester Hydrolase from Thermobifida Fusca. Biochemical Engineering Journal 2015, 93, 222–228. 10.1016/j.bej.2014.10.012.

[ref87] Barth M.; Wei R.; Oeser T.; Then J.; Schmidt J.; Wohlgemuth F.; Zimmermann W. Enzymatic Hydrolysis of Polyethylene Terephthalate Films in an Ultrafiltration Membrane Reactor. J. Membr. Sci. 2015, 494, 182–187. 10.1016/j.memsci.2015.07.030.

[ref88] Gao X.-Y.; Liu Y.; Miao L.-L.; Liu Z.-P. Pseudomonas Sp. AOB-7 Utilizes PHA Granules as a Sustained-Release Carbon Source and Biofilm Carrier for Aerobic Denitrification of Aquaculture Water. Appl. Microbiol. Biotechnol. 2020, 104 (7), 3183–3192. 10.1007/s00253-020-10452-y. 32055912

[ref89] Jeepery I. F.; Sudesh K.; Abe H. Miscibility and Enzymatic Degradability of Poly(3-Hydroxybutyrate-Co-3-Hydroxyhexanoate)-Based Polyester Blends by PHB Depolymerase and Lipase. Polym. Degrad. Stab. 2021, 192, 109692 10.1016/j.polymdegradstab.2021.109692.

[ref90] Ma Y.; Yao M.; Li B.; Ding M.; He B.; Chen S.; Zhou X.; Yuan Y. Enhanced Poly(Ethylene Terephthalate) Hydrolase Activity by Protein Engineering. Engineering 2018, 4 (6), 888–893. 10.1016/j.eng.2018.09.007.

[ref91] Herrero Acero E.; Ribitsch D.; Dellacher A.; Zitzenbacher S.; Marold A.; Steinkellner G.; Gruber K.; Schwab H.; Guebitz G. M. Surface Engineering of a Cutinase from *Thermobifida Cellulosilytica* for Improved Polyester Hydrolysis: Surface Engineering of a Cutinase. Biotechnol. Bioeng. 2013, 110 (10), 2581–2590. 10.1002/bit.24930. 23592055

[ref92] Mrigwani A.; Thakur B.; Guptasarma P. Counter-intuitive Enhancement of Degradation of Polyethylene Terephthalate through Engineering of Lowered Enzyme Binding to Solid Plastic. Proteins 2023, 91 (6), 807–821. 10.1002/prot.26468. 36629323

[ref93] Arnal G.; Anglade J.; Gavalda S.; Tournier V.; Chabot N.; Bornscheuer U. T.; Weber G.; Marty A. Assessment of Four Engineered PET Degrading Enzymes Considering Large-Scale Industrial Applications. ACS Catal. 2023, 13 (20), 13156–13166. 10.1021/acscatal.3c02922. 37881793 PMC10594578

[ref94] Cui Y.; Chen Y.; Sun J.; Zhu T.; Pang H.; Li C.; Geng W.-C.; Wu B. Computational Redesign of a Hydrolase for Nearly Complete PET Depolymerization at Industrially Relevant High-Solids Loading. Nat. Commun. 2024, 15 (1), 1417 10.1038/s41467-024-45662-9. 38360963 PMC10869840

[ref95] Liu L.; Huang W.-C.; Liu Y.; Li M. Diversity of Cellulolytic Microorganisms and Microbial Cellulases. International Biodeterioration & Biodegradation 2021, 163, 105277 10.1016/j.ibiod.2021.105277.

[ref96] Xu Q.; Resch M. G.; Podkaminer K.; Yang S.; Baker J. O.; Donohoe B. S.; Wilson C.; Klingeman D. M.; Olson D. G.; Decker S. R.; Giannone R. J.; Hettich R. L.; Brown S. D.; Lynd L. R.; Bayer E. A.; Himmel M. E.; Bomble Y. J. Dramatic Performance of *Clostridium Thermocellum* Explained by Its Wide Range of Cellulase Modalities. Sci. Adv. 2016, 2 (2), e1501254 10.1126/sciadv.1501254. 26989779 PMC4788478

[ref97] Hilden L.; Johansson G. Recent Developments on Cellulases and Carbohydrate-Binding Modules with Cellulose Affinity. Biotechnol. Lett. 2004, 26 (22), 1683–1693. 10.1007/s10529-004-4579-8. 15604820

[ref98] Wu S.; Wu S. Processivity and the Mechanisms of Processive Endoglucanases. Appl. Biochem. Biotechnol. 2020, 190 (2), 448–463. 10.1007/s12010-019-03096-w. 31378843

[ref99] Bayer E. A.; Belaich J.-P.; Shoham Y.; Lamed R. The Cellulosomes: Multienzyme Machines for Degradation of Plant Cell Wall Polysaccharides. Annu. Rev. Microbiol. 2004, 58 (1), 521–554. 10.1146/annurev.micro.57.030502.091022. 15487947

[ref100] Artzi L.; Bayer E. A.; Moraïs S. Cellulosomes: Bacterial Nanomachines for Dismantling Plant Polysaccharides. Nat. Rev. Microbiol 2017, 15 (2), 83–95. 10.1038/nrmicro.2016.164. 27941816

[ref101] Zverlov V. V.; Klupp M.; Krauss J.; Schwarz W. H. Mutations in the Scaffoldin Gene, *cipA*, of *Clostridium Thermocellum* with Impaired Cellulosome Formation and Cellulose Hydrolysis: Insertions of a New Transposable Element, IS 1447, and Implications for Cellulase Synergism on Crystalline Cellulose. J. Bacteriol. 2008, 190 (12), 4321–4327. 10.1128/JB.00097-08. 18408027 PMC2446765

[ref102] Eibinger M.; Ganner T.; Plank H.; Nidetzky B. A Biological Nanomachine at Work: Watching the Cellulosome Degrade Crystalline Cellulose. ACS Cent. Sci. 2020, 6 (5), 739–746. 10.1021/acscentsci.0c00050. 32490190 PMC7256933

[ref103] Final Report Summary - CELLULOSOMEPLUS (Boosting Lignocellulose Biomass Deconstruction with Designer Cellulosomes for Industrial Applications); CELLULOSOMEPLUS; grant agreement ID: 604530. https://cordis.europa.eu/project/id/604530/reporting.

[ref104] Lee K.; Jing Y.; Wang Y.; Yan N. A Unified View on Catalytic Conversion of Biomass and Waste Plastics. Nat. Rev. Chem. 2022, 6 (9), 635–652. 10.1038/s41570-022-00411-8. 37117711 PMC9366821

[ref105] Kasuya K.; Ohura T.; Masuda K.; Doi Y. Substrate and Binding Specificities of Bacterial Polyhydroxybutyrate Depolymerases. Int. J. Biol. Macromol. 1999, 24 (4), 329–336. 10.1016/S0141-8130(99)00046-X. 10408639

[ref106] Graham R.; Erickson E.; Brizendine R. K.; Salvachúa D.; Michener W. E.; Li Y.; Tan Z.; Beckham G. T.; McGeehan J. E.; Pickford A. R. The Role of Binding Modules in Enzymatic Poly(Ethylene Terephthalate) Hydrolysis at High-Solids Loadings. Chem. Catalysis 2022, 2 (10), 2644–2657. 10.1016/j.checat.2022.07.018.

[ref107] Thomsen T. B.; Hunt C. J.; Meyer A. S. Influence of Substrate Crystallinity and Glass Transition Temperature on Enzymatic Degradation of Polyethylene Terephthalate (PET). New Biotechnology 2022, 69, 28–35. 10.1016/j.nbt.2022.02.006. 35247624

[ref108] Xue R.; Chen Y.; Rong H.; Wei R.; Cui Z.; Zhou J.; Dong W.; Jiang M. Fusion of Chitin-Binding Domain From Chitinolyticbacter Meiyuanensis SYBC-H1 to the Leaf-Branch Compost Cutinase for Enhanced PET Hydrolysis. Front. Bioeng. Biotechnol. 2021, 9, 762854 10.3389/fbioe.2021.762854. 34976965 PMC8715031

[ref109] Hwang D.-H.; Lee M.-E.; Cho B.-H.; Oh J. W.; You S. K.; Ko Y. J.; Hyeon J. E.; Han S. O. Enhanced Biodegradation of Waste Poly(Ethylene Terephthalate) Using a Reinforced Plastic Degrading Enzyme Complex. Science of The Total Environment 2022, 842, 156890 10.1016/j.scitotenv.2022.156890. 35753492

[ref110] Knott B. C.; Erickson E.; Allen M. D.; Gado J. E.; Graham R.; Kearns F. L.; Pardo I.; Topuzlu E.; Anderson J. J.; Austin H. P.; Dominick G.; Johnson C. W.; Rorrer N. A.; Szostkiewicz C. J.; Copié V.; Payne C. M.; Woodcock H. L.; Donohoe B. S.; Beckham G. T.; McGeehan J. E. Characterization and Engineering of a Two-Enzyme System for Plastics Depolymerization. Proc. Natl. Acad. Sci. U.S.A. 2020, 117 (41), 25476–25485. 10.1073/pnas.2006753117. 32989159 PMC7568301

[ref111] Yan F.; Wei R.; Cui Q.; Bornscheuer U. T.; Liu Y. Thermophilic Whole-cell Degradation of Polyethylene Terephthalate Using Engineered *Clostridium Thermocellum*. Microbial Biotechnology 2021, 14 (2), 374–385. 10.1111/1751-7915.13580. 32343496 PMC7936307

[ref112] Ribitsch D.; Yebra A. O.; Zitzenbacher S.; Wu J.; Nowitsch S.; Steinkellner G.; Greimel K.; Doliska A.; Oberdorfer G.; Gruber C. C.; Gruber K.; Schwab H.; Stana-Kleinschek K.; Acero E. H.; Guebitz G. M. Fusion of Binding Domains to Thermobifida Cellulosilytica Cutinase to Tune Sorption Characteristics and Enhancing PET Hydrolysis. Biomacromolecules 2013, 14 (6), 1769–1776. 10.1021/bm400140u. 23718548

[ref113] Gomes D.; Matamá T.; Cavaco-Paulo A.; Takaki G.; Salgueiro A. Production of Heterologous Cutinases by E. Coli and Improved Enzyme Formulation for Application on Plastic Degradation. Electron. J. Biotechnol. 2013, 16 (5), 3 10.2225/vol16-issue5-fulltext-12.

[ref114] Ribitsch D.; Herrero Acero E.; Przylucka A.; Zitzenbacher S.; Marold A.; Gamerith C.; Tscheließnig R.; Jungbauer A.; Rennhofer H.; Lichtenegger H.; Amenitsch H.; Bonazza K.; Kubicek C. P.; Druzhinina I. S.; Guebitz G. M. Enhanced Cutinase-Catalyzed Hydrolysis of Polyethylene Terephthalate by Covalent Fusion to Hydrophobins. Appl. Environ. Microbiol. 2015, 81 (11), 3586–3592. 10.1128/AEM.04111-14. 25795674 PMC4421044

[ref115] Dai L.; Qu Y.; Huang J.-W.; Hu Y.; Hu H.; Li S.; Chen C.-C.; Guo R.-T. Enhancing PET Hydrolytic Enzyme Activity by Fusion of the Cellulose-Binding Domain of Cellobiohydrolase I from Trichoderma Reesei. J. Biotechnol. 2021, 334, 47–50. 10.1016/j.jbiotec.2021.05.006. 34044062

[ref116] Su L.; Chen K.; Bai S.; Yu L.; Sun Y. Cutinase Fused with C-Terminal Residues of α-Synuclein Improves Polyethylene Terephthalate Degradation by Enhancing the Substrate Binding. Biochemical Engineering Journal 2022, 188, 108709 10.1016/j.bej.2022.108709.

[ref117] Liu Z.; Zhang Y.; Wu J. Enhancement of PET Biodegradation by Anchor Peptide-Cutinase Fusion Protein. Enzyme Microb. Technol. 2022, 156, 110004 10.1016/j.enzmictec.2022.110004. 35217214

[ref118] Tian R.; Sun Y. α-Synuclein: A Fusion Chaperone Significantly Boosting the Enzymatic Performance of PET Hydrolase. Chinese Journal of Chemical Engineering 2023, 64, 18–25. 10.1016/j.cjche.2023.06.015.

[ref119] Kim H. T.; Hee Ryu M.; Jung Y. J.; Lim S.; Song H. M.; Park J.; Hwang S. Y.; Lee H.; Yeon Y. J.; Sung B. H.; Bornscheuer U. T.; Park S. J.; Joo J. C.; Oh D. X. Chemo-Biological Upcycling of Poly(Ethylene Terephthalate) to Multifunctional Coating Materials. ChemSusChem 2021, 14 (19), 4251–4259. 10.1002/cssc.202100909. 34339110 PMC8519047

[ref120] Xue R.; Qiu C.; Zhou X.; Cheng Y.; Zhang Z.; Zhang Y.; Schröder U.; Bornscheuer U. T.; Dong W.; Wei R.; Jiang M. Enzymatic Upcycling of PET Waste to Calcium Terephthalate for Battery Anodes. Angew. Chem. Int. Ed 2024, 63 (1), e202313633 10.1002/anie.202313633. 37880836

[ref121] Gopal M. R.; Dickey R. M.; Butler N. D.; Talley M. R.; Nakamura D. T.; Mohapatra A.; Watson M. P.; Chen W.; Kunjapur A. M. Reductive Enzyme Cascades for Valorization of Polyethylene Terephthalate Deconstruction Products. ACS Catal. 2023, 13 (7), 4778–4789. 10.1021/acscatal.2c06219.

[ref122] Qian X.; Jiang M.; Dong W. Tandem Chemical Deconstruction and Biological Upcycling of Poly(Ethylene Terephthalate). Trends Biotechnol. 2023, 41 (10), 1223–1226. 10.1016/j.tibtech.2023.03.021. 37105776

[ref123] Mudondo J.; Lee H.-S.; Jeong Y.; Kim T. H.; Kim S.; Sung B. H.; Park S.-H.; Park K.; Cha H. G.; Yeon Y. J.; Kim H. T. Recent Advances in the Chemobiological Upcycling of Polyethylene Terephthalate (PET) into Value-Added Chemicals. J. Microbiol. Biotechnol. 2023, 33 (1), 1–14. 10.4014/jmb.2208.08048. 36451300 PMC9895998

[ref124] Woo H.; Kang S. H.; Kwon Y.; Choi Y.; Kim J.; Ha D.-H.; Tanaka M.; Okochi M.; Kim J. S.; Kim H. K.; Choi J. Sensitive and Specific Capture of Polystyrene and Polypropylene Microplastics Using Engineered Peptide Biosensors. RSC Adv. 2022, 12 (13), 7680–7688. 10.1039/D1RA08701K. 35424716 PMC8982333

[ref125] Vodnik M.; Štrukelj B.; Lunder M. HWGMWSY, an Unanticipated Polystyrene Binding Peptide from Random Phage Display Libraries. Anal. Biochem. 2012, 424 (2), 83–86. 10.1016/j.ab.2012.02.013. 22370277

[ref126] Kumada Y.; Kuroki D.; Yasui H.; Ohse T.; Kishimoto M. Characterization of Polystyrene-Binding Peptides (PS-Tags) for Site-Specific Immobilization of Proteins. J. Biosci. Bioeng. 2010, 109 (6), 583–587. 10.1016/j.jbiosc.2009.11.005. 20471598

[ref127] Kumada Y.; Tokunaga Y.; Imanaka H.; Imamura K.; Sakiyama T.; Katoh S.; Nakanishi K. Screening and Characterization of Affinity Peptide Tags Specific to Polystyrene Supports for the Orientated Immobilization of Proteins. Biotechnol. Prog. 2006, 22 (2), 401–405. 10.1021/bp050331l. 16599553

[ref128] Yamada Y.; Anderson C. F.; Schneider J. P. De Novo Design of a Versatile Peptide-Based Coating to Impart Targeted Functionality at the Surface of Native Polystyrene. ACS Appl. Mater. Interfaces 2023, 15 (23), 27560–27567. 10.1021/acsami.3c02606. 37276244

[ref129] Bakhshinejad B.; Sadeghizadeh M. A Polystyrene Binding Target-Unrelated Peptide Isolated in the Screening of Phage Display Library. Anal. Biochem. 2016, 512, 120–128. 10.1016/j.ab.2016.08.013. 27555439

[ref130] Rübsam K.; Stomps B.; Böker A.; Jakob F.; Schwaneberg U. Anchor Peptides: A Green and Versatile Method for Polypropylene Functionalization. Polymer 2017, 116, 124–132. 10.1016/j.polymer.2017.03.070.

[ref131] Rübsam K.; Davari M.; Jakob F.; Schwaneberg U. KnowVolution of the Polymer-Binding Peptide LCI for Improved Polypropylene Binding. Polymers 2018, 10 (4), 423 10.3390/polym10040423. 30966458 PMC6415234

[ref132] Rübsam K.; Weber L.; Jakob F.; Schwaneberg U. Directed Evolution of Polypropylene and Polystyrene Binding Peptides. Biotechnol. Bioeng. 2018, 115 (2), 321–330. 10.1002/bit.26481. 29064564

[ref133] Jin J.; Arciszewski J.; Auclair K.; Jia Z. Enzymatic Polyethylene Biorecycling: Confronting Challenges and Shaping the Future. Journal of Hazardous Materials 2023, 460, 132449 10.1016/j.jhazmat.2023.132449. 37690195

[ref134] Fenibo E. O.; Selvarajan R.; Abia A. L. K.; Matambo T. Medium-Chain Alkane Biodegradation and Its Link to Some Unifying Attributes of alkB Genes Diversity. Science of The Total Environment 2023, 877, 162951 10.1016/j.scitotenv.2023.162951. 36948313

[ref135] Kong D.; Wang L.; Chen X.; Xia W.; Su L.; Zuo F.; Yan Z.; Chen S.; Wu J. Chemical-Biological Degradation of Polyethylene Combining Baeyer-Villiger Oxidation and Hydrolysis Reaction of Cutinase. Green Chem. 2022, 24 (5), 2203–2211. 10.1039/D2GC00425A.

[ref136] Von Haugwitz G.; Donnelly K.; Di Filippo M.; Breite D.; Phippard M.; Schulze A.; Wei R.; Baumann M.; Bornscheuer U. T. Synthesis of Modified Poly(Vinyl Alcohol)s and Their Degradation Using an Enzymatic Cascade. Angew. Chem. Int. Ed 2023, 62 (18), e202216962 10.1002/anie.202216962. 36637456

[ref137] Fürst M. J. L. J.; Gran-Scheuch A.; Aalbers F. S.; Fraaije M. W. Baeyer-Villiger Monooxygenases: Tunable Oxidative Biocatalysts. ACS Catal. 2019, 9 (12), 11207–11241. 10.1021/acscatal.9b03396.

[ref138] Yeletsky P. M.; Kukushkin R. G.; Yakovlev V. A.; Chen B. H. Recent Advances in One-Stage Conversion of Lipid-Based Biomass-Derived Oils into Fuel Components - Aromatics and Isomerized Alkanes. Fuel 2020, 278, 118255 10.1016/j.fuel.2020.118255. 32834073 PMC7313509

[ref139] Karis D.; Cain R.; Young K.; Shand A.; Holm T.; Springer E. Non-fuel Uses for Fatty Acid Methyl Esters. Biofuels Bioprod Bioref 2022, 16 (6), 1893–1908. 10.1002/bbb.2422.

[ref140] De Gonzalo G.; Paul C. E. Recent Trends in Synthetic Enzymatic Cascades Promoted by Alcohol Dehydrogenases. Current Opinion in Green and Sustainable Chemistry 2021, 32, 100548 10.1016/j.cogsc.2021.100548.

[ref141] Shortall K.; Djeghader A.; Magner E.; Soulimane T. Insights into Aldehyde Dehydrogenase Enzymes: A Structural Perspective. Front. Mol. Biosci. 2021, 8, 659550 10.3389/fmolb.2021.659550. 34055881 PMC8160307

[ref142] Fang B.; Xu H.; Liu Y.; Qi F.; Zhang W.; Chen H.; Wang C.; Wang Y.; Yang W.; Li S. Mutagenesis and Redox Partners Analysis of the P450 Fatty Acid Decarboxylase OleTJE. Sci. Rep 2017, 7 (1), 44258 10.1038/srep44258. 28276499 PMC5343568

[ref143] Seo M.-J.; Schmidt-Dannert C. Organizing Multi-Enzyme Systems into Programmable Materials for Biocatalysis. Catalysts 2021, 11 (4), 409 10.3390/catal11040409.

[ref144] Xie S.; Qiu X.; Zhu L.; Zhu C.; Liu C.; Wu X.; Zhu L.; Zhang D. Assembly of TALE-Based DNA Scaffold for the Enhancement of Exogenous Multi-Enzymatic Pathway. J. Biotechnol. 2019, 296, 69–74. 10.1016/j.jbiotec.2019.03.008. 30885657

[ref145] Fu J.; Yang Y. R.; Johnson-Buck A.; Liu M.; Liu Y.; Walter N. G.; Woodbury N. W.; Yan H. Multi-Enzyme Complexes on DNA Scaffolds Capable of Substrate Channelling with an Artificial Swinging Arm. Nat. Nanotechnol. 2014, 9 (7), 531–536. 10.1038/nnano.2014.100. 24859813

[ref146] Wang X.; Jiang Y.; Liu H.; Yuan H.; Huang D.; Wang T. Research Progress of Multi-Enzyme Complexes Based on the Design of Scaffold Protein. Bioresour. Bioprocess. 2023, 10 (1), 72 10.1186/s40643-023-00695-8. 38647916 PMC10992622

[ref147] Dubey N. C.; Tripathi B. P. Nature Inspired Multienzyme Immobilization: Strategies and Concepts. ACS Appl. Bio Mater. 2021, 4 (2), 1077–1114. 10.1021/acsabm.0c01293. 35014469

[ref148] Ellis G. A.; Klein W. P.; Lasarte-Aragonés G.; Thakur M.; Walper S. A.; Medintz I. L. Artificial Multienzyme Scaffolds: Pursuing *in Vitro* Substrate Channeling with an Overview of Current Progress. ACS Catal. 2019, 9 (12), 10812–10869. 10.1021/acscatal.9b02413.

[ref149] Liu Z.; Cao S.; Liu M.; Kang W.; Xia J. Self-Assembled Multienzyme Nanostructures on Synthetic Protein Scaffolds. ACS Nano 2019, 13 (10), 11343–11352. 10.1021/acsnano.9b04554. 31498583

[ref150] Wang S.-Z.; Zhang Y.-H.; Ren H.; Wang Y.-L.; Jiang W.; Fang B.-S. Strategies and Perspectives of Assembling Multi-Enzyme Systems. Critical Reviews in Biotechnology 2017, 37 (8), 1024–1037. 10.1080/07388551.2017.1303803. 28423958

[ref151] Fontes C. M. G. A.; Gilbert H. J. Cellulosomes: Highly Efficient Nanomachines Designed to Deconstruct Plant Cell Wall Complex Carbohydrates. Annu. Rev. Biochem. 2010, 79 (1), 655–681. 10.1146/annurev-biochem-091208-085603. 20373916

[ref152] Gunnoo M.; Cazade P.; Galera-Prat A.; Nash M. A.; Czjzek M.; Cieplak M.; Alvarez B.; Aguilar M.; Karpol A.; Gaub H.; Carrión-Vázquez M.; Bayer E. A.; Thompson D. Nanoscale Engineering of Designer Cellulosomes. Adv. Mater. 2016, 28 (27), 5619–5647. 10.1002/adma.201503948. 26748482

[ref153] Stern J.; Moraïs S.; Lamed R.; Bayer E. A. Adaptor Scaffoldins: An Original Strategy for Extended Designer Cellulosomes, Inspired from Nature. mBio 2016, 7 (2), e00083 10.1128/mBio.00083-16. 27048796 PMC4959524

[ref154] Aer L.; Jiang Q.; Zhong L.; Si Q.; Liu X.; Pan Y.; Feng J.; Zeng H.; Tang L. Optimization of Polyethylene Terephthalate Biodegradation Using a Self-Assembled Multi-Enzyme Cascade Strategy. Journal of Hazardous Materials 2024, 476, 134887 10.1016/j.jhazmat.2024.134887. 38901251

[ref155] Bugada L. F.; Smith M. R.; Wen F. Engineering Spatially Organized Multienzyme Assemblies for Complex Chemical Transformation. ACS Catal. 2018, 8 (9), 7898–7906. 10.1021/acscatal.8b01883.

[ref156] Woodley J. M. Enzyme Cascade Process Design and Modelling. In Enzyme Cascade Design and Modelling; Kara S.; Rudroff F., Eds.; Springer International Publishing: Cham, 2021; pp 125–139. 10.1007/978-3-030-65718-5_8.

[ref157] Song Z.; Zhang Q.; Wu W.; Pu Z.; Yu H. Rational Design of Enzyme Activity and Enantioselectivity. Front. Bioeng. Biotechnol. 2023, 11, 1129149 10.3389/fbioe.2023.1129149. 36761300 PMC9902596

[ref158] Schwarz W. H. The Cellulosome and Cellulose Degradation by Anaerobic Bacteria. Appl. Microbiol. Biotechnol. 2001, 56 (5–6), 634–649. 10.1007/s002530100710. 11601609

